# SMTracker: a tool for quantitative analysis, exploration and visualization of single-molecule tracking data reveals highly dynamic binding of *B*. *subtilis* global repressor AbrB throughout the genome

**DOI:** 10.1038/s41598-018-33842-9

**Published:** 2018-10-24

**Authors:** Thomas C. Rösch, Luis M. Oviedo-Bocanegra, Georg Fritz, Peter L. Graumann

**Affiliations:** 1 SYNMIKRO, LOEWE Center for Synthetic Microbiology, Marburg, Germany; 2 Department of Chemistry, Philipps Universität Marburg, Marburg, Germany; 3 Department of Physics, Philipps Universität Marburg, Marburg, Germany

## Abstract

Single-particle (molecule) tracking (SPT/SMT) is a powerful method to study dynamic processes in living cells at high spatial and temporal resolution. Even though SMT is becoming a widely used method in bacterial cell biology, there is no program employing different analytical tools for the quantitative evaluation of tracking data. We developed SMTracker, a MATLAB-based graphical user interface (GUI) for automatically quantifying, visualizing and managing SMT data via five interactive panels, allowing the user to interactively explore tracking data from several conditions, movies and cells on a track-by-track basis. Diffusion constants are calculated a) by a Gaussian mixture model (GMM) panel, analyzing the distribution of positional displacements in *x-* and *y-*direction using a multi-state diffusion model (e.g. DNA-bound vs. freely diffusing molecules), and inferring the diffusion constants and relative fraction of molecules in each state, or b) by square displacement analysis (SQD), using the cumulative probability distribution of square displacements to estimate the diffusion constants and relative fractions of up to three diffusive states, or c) through mean-squared displacement (MSD) analyses, allowing the discrimination between Brownian, sub- or superdiffusive behavior. A spatial distribution analysis (SDA) panel analyzes the subcellular localization of molecules, summarizing the localization of trajectories in 2D- heat maps. Using SMTracker, we show that the global transcriptional repressor AbrB performs highly dynamic binding throughout the *Bacillus subtilis* genome, with short dwell times that indicate high on/off rates *in vivo*. While about a third of AbrB molecules are in a DNA-bound state, 40% diffuse through the chromosome, and the remaining molecules freely diffuse through the cells. AbrB also forms one or two regions of high intensity binding on the nucleoids, similar to the global gene silencer H-NS in *Escherichia coli*, indicating that AbrB may also confer a structural function in genome organization.

## Introduction

With the advent of single-molecule localization microscopy, there is an increasing number of studies reporting the dynamics of single molecules at the millisecond range and at high optical resolution^1^. Contrarily to ensemble measurements, such as fluorescence correlation spectroscopy (FCS) and fluorescence recovery after photobleaching (FRAP) experiments, single molecule tracking (SMT) provides details about individual molecules and their diffusive behavior^2^ and has revealed unprecedented insights into the mechanism of diverse cellular processes such as signal transduction^3^, chromosome segregation^4,5^, transcription^6^, translation^7,8^, replication^9^ and DNA-repair^10–12^.

Despite several established software solutions to detect and connect sequentially acquired signals of fluorescent fusion proteins^13^, we are aware of only few GUI-based tools to easily asses, visualize and further analyze diffusion data without advanced computational skills. The softwares InferenceMAP and Diatrack^14,15^ are able to infer diffusion constants from tracking data and allows the user to visualize spatial maps of protein mobilities, primarily designed for eukaryotic cells. We therefore devised a GUI-based solution to easily quantify, explore and visualize the molecular kinetics and dynamics of single particles in bacterial cells. This program fills a void for tools that provide access to different diffusive states of individual molecules, as often incurred by binding and unbinding to other subcellular structures. We therefore developed SMTracker, a versatile and user-friendly program operating in MATLAB, which can be run without prior programming knowledge. The software allows detailed access to single trajectories, to analyze the mode of diffusion via different approaches, the determination of binding kinetics (e.g. of protein-DNA interactions) and the 2-dimensional visualization of subcellular distributions of molecules and their tracks in a normalised cell.

**Figure 1:**
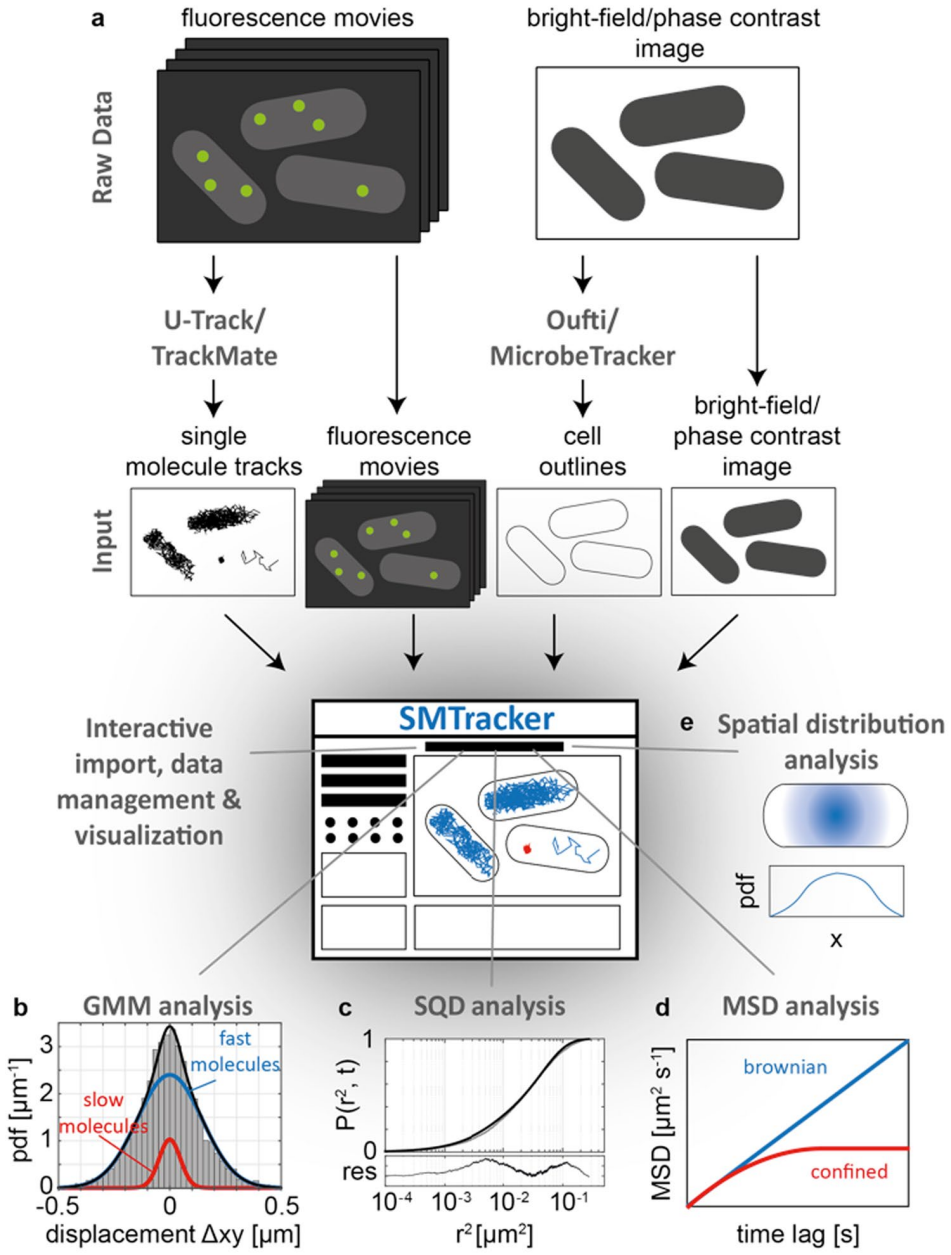
The SMTracker software suite. (**a**) After importing raw data of tracks and cell outlines, the import panel allows the user to interactively explore the data, identify characteristic patterns and potential experimental artifacts. (**b**) The Gaussian mixture model (GMM) analysis runs a fit on the probability density function (pdf) of positional 1D displacements, assuming a two- (or three-) state model with a mobile (blue line) and immobile (red line) subpopulation of molecules, thereby extracting the diffusion coefficient and fraction size of each subpopulation. (**c**) Squared displacement (SQD) analysis considers the cumulative probability function of squared displacements and provides an alternative way to analyze up to 3 different subpopulations in the biological sample. (**d**) The mean-squared displacement (MSD) analysis plots the time-ensemble averaged MSD vs. the time-lag, revealing the type of motion exhibited by the molecules. A linear dependency of the MSD on the time-lag indicates Brownian diffusion (blue line), whereas an asymptotic curve is indicative of confined (or sub-diffusive) motion. (**e**) In the spatial distribution analysis (SDA) panel the software generates 2D- and 3D- heat maps and distributions of trajectories along the *x-* and *y-*axis of the cell, allowing the identification of specific localization patterns of molecules, e.g., cytoplasmic, membrane- or nucleoid-bound.

SMTracker contains 5 different panels (Fig. 1): (i) The import/exploration panel allows the user to interactively explore tracking data from several conditions, movies and cells on a track-by-track basis. (ii) The Gaussian mixture model (GMM) panel analyzes the distribution of positional displacements in *x-* and *y-*direction using a multi-state diffusion model (e.g. DNA-bound vs. freely diffusing molecules), and infers the diffusion constants and relative fraction of molecules in each state. (iii) The squared displacement analysis (SQD) panel uses the cumulative probability distribution of squared displacements to estimate the diffusion constants and relative fractions of up to three diffusive states. Although the GMM and SQD analyses are closely related, each method has strengths and weaknesses in accurately inferring molecular properties under different experimental conditions, e.g. for different magnitudes of the diffusion constant, as shown by benchmarking both methods on computer-simulated SMT data. (iv) The mean-square displacement (MSD) panel determines the mode of diffusion from a fit of the MSD plotted against the time lag between image frames, allowing the discrimination between Brownian, sub- or superdiffusive behavior. (v) The spatial distribution analysis (SDA) panel analyzes the subcellular localization of molecules, summarizing the localization of trajectories in 2D- and 3D-heat maps and in normalized distributions projected in *x-* and *y-*orientation. This visualization allows distinguishing different localization patterns (cytoplasmic, nucleoid- or membrane-bound) arising from different biological conditions.

AbrB is a tetrameric DNA binding protein that generally represses many genes during exponential growth, which are released from repression at the transition to stationary phase, rendering AbrB an important global regulator for stationary phase gene expression (including biofilm formation) in *Bacillus subtilis* and other Gram positive bacteria^16–18^. In many different bacterial species, including Cyanobacteria, AbrB confers an important function in gene regulation for e.g. metabolic processes, extending its activity into the exponential growth phase^19,20^. In *B*. *subtilis*, AbrB also binds to DNA as a heterotetramer, together with its paralog Abh, to a total of over 600 binding sites throughout the genome^21^. Of these, about 100 have been implicated in regulating promoter activities in a direct manner, which influences the expression of almost 200 genes. In spite of its genome-wide importance, and role as transition-state regulator, AbrB has never been characterized in a cell biological approach. Using SMTracker, we determined single molecule dynamics of AbrB, and also found a surprising clustering of AbrB molecules on the nucleoids that may confer a more general role as chromosome-structuring element.

## Results and Discussion

### Import/exploratory panel

As an input, SMTracker requires single-molecule tracking data, cell contour data and original fluorescence movies (Figs. 1a and S1). SMTracker supports data input from two of the most commonly used particle tracking tools U-track^22^ and TrackMate^23^ and computes the *x-* and *y-*coordinates of molecular trajectories relative to the geometry of each cell, as obtained by the cell segmentation tools MicrobeTracker^24^ or Oufti^25^. After automatic data import – only requiring the pixel size of the microscopy system and the time interval ${\rm{\Delta }}t$ between image frames as user input – each track is associated to a corresponding cell and the user can interactively explore tracking data from several conditions, movies and cells on a track-by-track basis. Cells and tracks can be manually removed, if necessary, and overlaid in up to three different channels acquired during imaging (e.g. DAPI, membrane or phase contrast), allowing for a manual analysis of protein localization relative to cellular markers. Besides choosing a specific trajectory or cell from a pop-up menu, the user can drag each cell or trajectory on the screen (Fig. S4) and thereby access statistical details about the track (Fig. S5) and visualize the track as a movie. Additionally, this panel provides general statistical information about the experiment (e.g. the number of movies, cells, trajectories, etc.) and the conditions studied.

### Gaussian mixture model (GMM) analysis panel

To directly compare the change in intracellular protein mobility upon changing conditions of the cell (e.g. drug treatment), we developed a method based on Gaussian mixture models (GMM). The method assumes that molecules may exist in multiple states, e.g. DNA-bound vs. free, or septum-bound and freely diffusive, each of which is associated with a different diffusion constant $${D}_{1}$$ and $${D}_{2}$$, respectively, and that the fraction $$\alpha $$ of molecules in the slow diffusive state (or $$1-\alpha $$ in the fast diffusive state) may change between experimental conditions (Fig. 1b). For each subpopulation the random motion between consecutive image frames leads to positional displacements in *x-* and *y-*direction, $${\rm{\Delta }}x$$ and $${\rm{\Delta }}y$$, following a Gaussian distribution with zero mean and standard deviation $$\sigma =\,\sqrt{2D{\rm{\Delta }}t}$$, where $$D$$ is the respective (1-dimensional) diffusion constant and $${\rm{\Delta }}t$$ the time interval between image frames. By fitting the superposition of one, two or optionally three Gaussians to the experimental displacement distributions the algorithm infers the diffusion constants $${D}_{1}$$, $${D}_{2}$$ (and $${D}_{3})$$ as well as the fractions $${\alpha }_{1},\,{\alpha }_{2}$$ and $${\alpha }_{3}$$ of the respective molecules in the different diffusive states. In particular, if we let

$$F({\rm{\Delta }}x|\,\mu ,\sigma )=\frac{1}{2}\,[1+{\rm{erf}}(\frac{{\rm{\Delta }}x-\mu }{\sqrt{2}\sigma })]$$

be the cumulative distribution function (CDF) of the Gaussian distribution, we performed a least square fit of the function

$$G({\rm{\Delta }}x|\alpha ,{D}_{1},{D}_{2})=\sum _{i=1}^{q}{\alpha }_{i}F({\rm{\Delta }}x|0,\sqrt{2{D}_{i}{\rm{\Delta }}t}),\,with\,\sum _{i=1}^{q}{\alpha }_{i}=1,$$

to the experimental CDF, $H({\rm{\Delta }}x),$ by using a trust-region reflective Newton method (MATLAB, The MathWorks, Inc.). Note that the algorithm allows the user to either pool *x-* and *y-*displacements into a single distribution *H* (which is the preferred choice if *x-* and *y-*displacements are given in an image-centric coordinate system and cells are randomly oriented) or to treat *x-* and *y-*displacements independently (which is the preferred choice if the analysis is performed in a cell-centric coordinate system, with the *x*- and *y-*axis corresponding to the long and the short cell axis, respectively). To evaluate the goodness of fit, a Kolmogorov-Smirnov test has been applied.

An example of a protein investigated by GMM can be seen in suppl. Fig. S6: in the left panel, many small steps (around “0”) can be seen, and fewer larger steps (these are shown by the dotted line), while in the right panel, the protein has become much more mobile; now, the static fraction (dotted line) is much smaller than the one in the left panel. The red curves show two population fits, which explain the data well, while a single fit (assuming a single population), shown in green, could not explain the observed distribution of steps. The bubble plot GMM (Fig. S6) shows the size of the two populations, and their average diffusion constant on the *y*-axis. It can be seen that the right condition has an inverse relation between static and mobile molecules, revealing that the protein markedly changes its dynamics between the two conditions.

To better compare changes in molecular behavior between different treatments of cells, the algorithm optionally allows the user to keep the diffusion constants ${D}_{1}$, ${D}_{2}$ (and ${D}_{3}$) identical between conditions and to attribute changes in the displacement distributions only to changes in the fractions ${\alpha }_{i}$. A two-sample Kolmogorov-Smirnov test has been implemented in order to evaluate significant differences between the experimental cumulative distributions of displacements. The software also runs a model comparison test based on the Bayesian Information Criterion (BIC)^26^, and displays which model (i.e. the number of diffusive states), explains the experimental data best, i.e., the model for which

$$BIC={\chi }^{2}+k\cdot \,\mathrm{ln}(n)$$

is minimal. Here, ${\chi }^{2}$ denotes the residual between model and experimental CDF for the optimal parameter set, $k$ the number of model parameters and $n$ the number of experimental data points.

As an additional output the software also calculates the residence times for the molecules of each condition, e.g. how long a protein binds to DNA, by counting the time a molecule stays within a predefined radius $R$. This radius is based on the standard deviation of the immobile fraction of molecules, ${\sigma }_{1}=\sqrt{2{D}_{1}{\rm{\Delta }}t},\,\,$such that it includes 99.7% of the smaller displacements ($R=3\cdot {\sigma }_{1}$), thereby also allowing the classification into confined and non-confined trajectories.

### Mean-square displacement (MSD) analysis panel

The most common way of analyzing SMT data is the extraction of the time-ensemble averaged MSD, which is plotted as a function of the time lag (*t*_*lag*_)^27^. While normal (Brownian) diffusion shows linear relation between the MSD and *t*_*lag*,_ confined motion is represented by a MSD that reaches a plateau for high *t*_*lag*_ and directed motion is characterized by parabolic MSD curve (Fig. 1d). By fitting the MSD plot with a linear function at short time-lags (Fig. S7), the diffusion coefficient can be estimated^28^. To this end the user can interactively define the number of *t*_*lag*_ taken into account to linearly fit the MSD function, which condition and which direction (*x-*, *y-* or pooled *xy-*orientation) should be plotted. Additionally, the panel provides a measure for the localization error $\xi$ of the microscopy system, which is derived from the *y*-axis intercept of the time-averaged MSD curves generated for single trajectories^28^. To improve precision in the estimation of $\xi$ the software limits this analysis to tracks with a high goodness of fit (R^2^) value and a low diffusion coefficient.

Here, it should be noted that the MSD analysis is most appropriate if individual molecules are in one diffusive state characterized by a single diffusion constant. However, if individual molecules switch between diffusive states (e.g. by DNA binding and unbinding), the MSD analysis will only report an average diffusion constant for each molecule. Thus, even if molecules are in clearly distinct diffusive states, but if they switch between them, the time-averaging performed on each trajectory can lead to a whole spectrum of apparent diffusion constants between ${D}_{1}$ and ${D}_{2}$.

### Squared displacement (SQD) analysis panel

The SQD panel integrates an alternative way of analyzing the diffusive behavior of the molecules at the sub-population level (Fig. 1c), which was first described by Schütz *et al*^29^. and has been applied in a variety of studies^5,30,31^. The basic idea is similar to the GMM analysis, but the SQD analysis considers the solution of the diffusion equation in terms of the 2-dimensional radial displacement $r{(t)}^{2}={(x(t)-x(0))}^{2}+{(y(t)-y(0))}^{2}$, which leads for one diffusive molecule species to the cumulative distribution function

$$P({r}^{2},t)=1-\exp (-\frac{{r}^{2}}{\sigma {(t)}^{2}})\,,$$

with

$$\sigma (t)=\sqrt{4Dt}\,.$$

Accordingly, for multiple diffusive species with diffusion constants ${D}_{i}\,(i=1,\ldots ,q)$ and relative fractions ${\alpha }_{i}$ the CDF reads

1 $$P({r}^{2},t)=1-\sum _{i=1}^{q}{\alpha }_{i}\cdot (\exp (-\frac{{r}^{2}}{4{D}_{i}t}))\,,$$

where $\sum _{i=1}^{q}{\alpha }_{i}=1$. For a given number of diffusive states $q$, the algorithm implemented in the SQD panel performs a simultaneous nonlinear least square fit of Eq. (1) to the experimental CDFs for $t=1{\rm{\Delta }}t,\,2{\rm{\Delta }}t,\,3{\rm{\Delta }}t,4{\rm{\Delta }}t$ - thereby estimating the ${\alpha }_{i}$ and ${D}_{i}$^32^. Here, the user can either choose manually between a one-, two- or three-state model to estimate the ${\alpha }_{i}$ and ${D}_{i}$ of a given dataset, or to automatically choose the best-fitting model according to the BIC criterion (Fig. S8). Please note that in contrast to the GMM method, the CDF of squared displacements are fitted independently for each condition or treatment. For the evaluation of the difference between the CDFs of the modelled distributions of each pair of conditions, a two-sample Kolmogorov-Smirnov is performed.

### Spatial distribution (SDA) panel

The spatial distribution panel summarizes the localization of the trajectories in 2D- and 3D-heat maps and provides normalized distributions for the localization in *x*- and *y*-orientation. To this end all trajectories are transformed into a cell-centric coordinate system and then scaled to a unit cell of $1\,\mu m\,\times \,1\,\mu m$ (Fig. S9). This visualization allows distinguishing different localization patterns (cytoplasmic, nucleoid and membrane) (Fig. 1e), and the visualization of changes in localization patterns of a given protein in response to e.g. changes in environmental conditions, or by the absence of another protein thought to interact with the protein of interest.

### Benchmarking SMTracker performance with synthetic SMT data

In order to validate the performance of SMTracker, we used computational simulations to generate sets of synthetic SMT data with known properties, i.e., diffusion constants and fraction sizes of molecules in the different diffusive states. Applying the GMM and SQD methods to the synthetic data then allows benchmarking the performance of each method as a function of simulation parameters, as detailed in the following.

### Simulation of synthetic single molecule trajectories

Simulations of single molecule trajectories were performed on a total of ${n}_{tot}=\,{n}_{1}+{n}_{2}$ molecules, where ${n}_{1}$ and ${n}_{2}$ are the number of molecules in a slow and fast diffusive state (with diffusion constants ${D}_{1}$ and ${D}_{2}$), respectively. For each subpopulation, Brownian motion of molecules was modeled by a 3-dimensional discrete-time random walk in *x-*, *y-*, and *z-*direction according to

$${x}_{i+1}={x}_{i}+{\delta }_{x};\,{\delta }_{x}\sim N(0,\sqrt{2D\tau })$$

$${y}_{i+1}={y}_{i}+{\delta }_{y};\,{\delta }_{y}\sim N(0,\sqrt{2D\tau })$$

$${z}_{i+1}={z}_{i}+{\delta }_{z};\,{\delta }_{z}\sim N(0,\sqrt{2D\tau })\,{\rm{.}}$$

Here, ${\delta }_{x},\,{\delta }_{y}$ and ${\delta }_{z}$ are Gaussian-distributed random variables with zero mean and standard deviation $\sigma =\sqrt{2D\tau }\,$, with $D\,\in \,\{{D}_{1},{D}_{2}\}$ being the diffusion constant of the respective subpopulation and $\tau$ the time interval between simulation steps $i=\,1,\,2,\ldots ,{N}_{t}$. The initial particle positions $({x}_{0},{y}_{0},{z}_{0})$ were randomly sampled according to a uniform distribution within a 3-dimensional, rod-shaped cell, formed by a cylinder and two hemispherical end caps with a radius of $0.5\,\mu m\,\,$and a total length of $3\,\mu m$. From these starting points tridimensional random trajectories were simulated with a time lag of $\tau =0.02\,ms$. Reflecting boundary conditions at the cell membrane were implemented by rejecting (and re-drawing) all random moves (${\delta }_{x},{\delta }_{y},{\delta }_{z}$) leading to positional vectors outside the rod-shaped cell. Additionally, the simulation accounts for a finite localization precision in typical experimental setups by adding a random vector (${\varepsilon }_{x},{\varepsilon }_{y},{\varepsilon }_{z})$ to the “true” positions in the simulation, leading to the “observed” positions $({X}_{i},{Y}_{i},{Z}_{i})$ at each point in time $i$

$${X}_{i}={x}_{i}+{\varepsilon }_{x};\,{\varepsilon }_{x}\sim N(0,{{\rm{\sigma }}}_{x,loc})$$

$${Y}_{i}={y}_{i}+{\varepsilon }_{y};\,{\varepsilon }_{y}\sim N(0,{{\rm{\sigma }}}_{y,loc})$$

$${Z}_{i}={z}_{i}+{\varepsilon }_{z};\,{\varepsilon }_{z}\sim N(0,{{\rm{\sigma }}}_{z,loc}).$$

here the (${\varepsilon }_{x},{\varepsilon }_{y},{\varepsilon }_{z})$ are again normally distributed random numbers with zero mean and standard deviation ${{\rm{\sigma }}}_{x,loc}=\,{{\rm{\sigma }}}_{y,loc}={{\rm{\sigma }}}_{x,loc}={{\rm{\sigma }}}_{loc}=30\,nm$. For the generation of synthetic single molecule trajectories, we used a sampling interval of ${\rm{\Delta }}t=20\,ms$, which is often used in experimental setups (see Tables 1 and 2 for a summary of all simulation parameters).

**Table 1:** Configuration of the synthetic trajectories.

Number of tracks	2000
Max number of points/track	20
Simulation time lag *τ* (ms)	0.02
Observation time lag (ms)	20
Number of simulation runs	30
Diffusion constant *D*_1_ (μm^2^ s^−1^)	[0.01 0.05 0.1]
Diffusion constant *D*_2_ (μm^2^ s^−1^)	[0.1 0.5 1]
Fraction size $\alpha$ (% molecules at diffusion rate *D*_1_)	[20 40 60 80]
Localization error $\xi \,$(nm)	30

**Table 2:** Configuration of the fitting procedure.

Fitting upper boundaries [*D*_1_, *D*_2_] (μm^2^ s^−1^)	[1, 10]
Fitting lower boundaries [*D*_1_, *D*_2_] (μm^2^ s^−1^)	[0.0225, 0.0225]
Fit method	Nonlinear least-squares
Tolerance	10^−8^

### Benchmarking of GMM and SQD method performance

**Figure 2:**
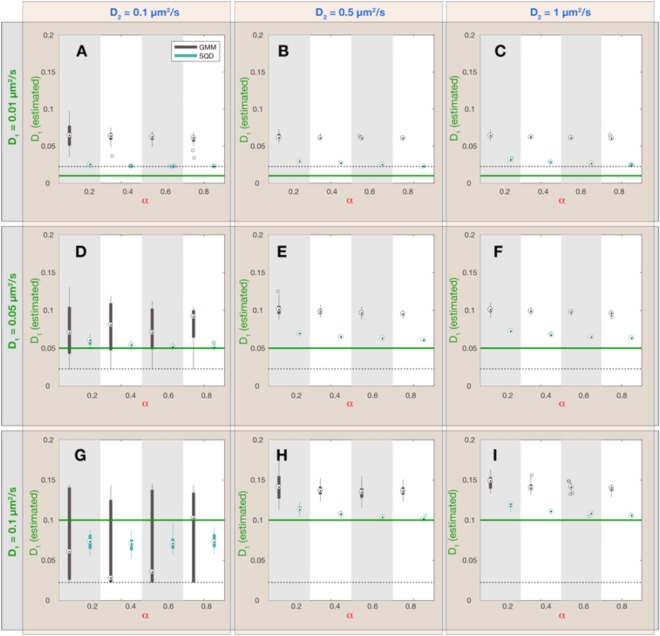
Dependence of estimated diffusion constant ${{D}}_{1}$ (slow subpopulation) on simulation parameters and inference method. Each panel (A–I) corresponds to a combination of simulation parameters ${D}_{1}$ and ${D}_{2}$, as indicated by the green and blue labels outside the plot panels, respectively. Within each panel 4 values of the fraction sizes $\alpha$ were chosen and synthetic simulations were performed on all parameter combinations. For each parameter combination (${D}_{1}$, ${D}_{2}$ and $\alpha$) 30 simulations with 2000 tracks of 20 frames duration (see Table 1 for all simulation parameters) were executed and the resulting data was analyzed with the GMM and SQD method, yielding two different estimates for the value of ${D}_{1}$(GMM: black boxplots; SQD: cyan boxplots). On each box, the central mark is the median, the edges of the box are the 25^th^ and 75^th^ percentiles, the whiskers extend to the most extreme data points not considered outliers and the outliers are plotted as individual circles. The green horizontal lines indicate the true value for ${D}_{1}\,$in the simulation. The black dashed lines indicate the lower limit ${D}_{loc}=\frac{{{\rm{\sigma }}}_{loc}^{2}}{2{\rm{\Delta }}t}=0.0225\frac{\mu {m}^{2}}{s}$ for an estimate of the diffusion constant, given a finite localization precision ${{\rm{\sigma }}}_{loc}=30\,nm$ and a frame rate of ${\rm{\Delta }}t=20\,ms$ used in our simulations.

**Figure 3:**
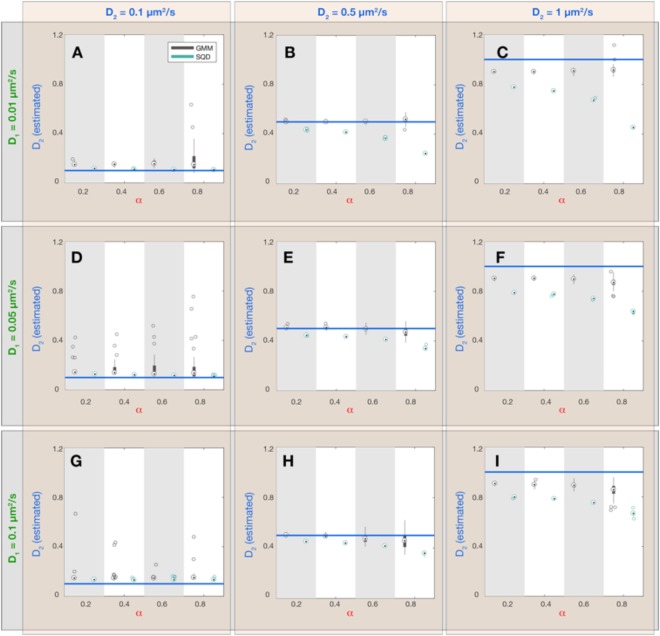
Dependence of estimated diffusion constant ${{D}}_{2}$ (fast subpopulation) on simulation parameters and inference method. Each panel (A–I) corresponds to a combination of simulation parameters ${D}_{1}$ and ${D}_{2}$, as indicated by the green and blue labels outside the plot panels, respectively. Within each panel 4 values of the fraction sizes $\alpha$ were chosen and synthetic simulations were performed on all parameter combinations. For each parameter combination (${D}_{1}$, ${D}_{2}$ and $\alpha$) 30 simulations with 2000 tracks of 20 frames duration (see Table 1 for all simulation parameters) were executed and the resulting data was analyzed with the GMM and SQD method, yielding two different estimates for the value of ${D}_{2}$(GMM: black boxplots; SQD: cyan boxplots). On each box, the central mark is the median, the edges of the box are the 25^th^ and 75^th^ percentiles, the whiskers extend to the most extreme data points not considered outliers and the outliers are plotted as individual circles. The blue horizontal lines indicate the true value for ${D}_{2}\,$in the simulation.

**Figure 4:**
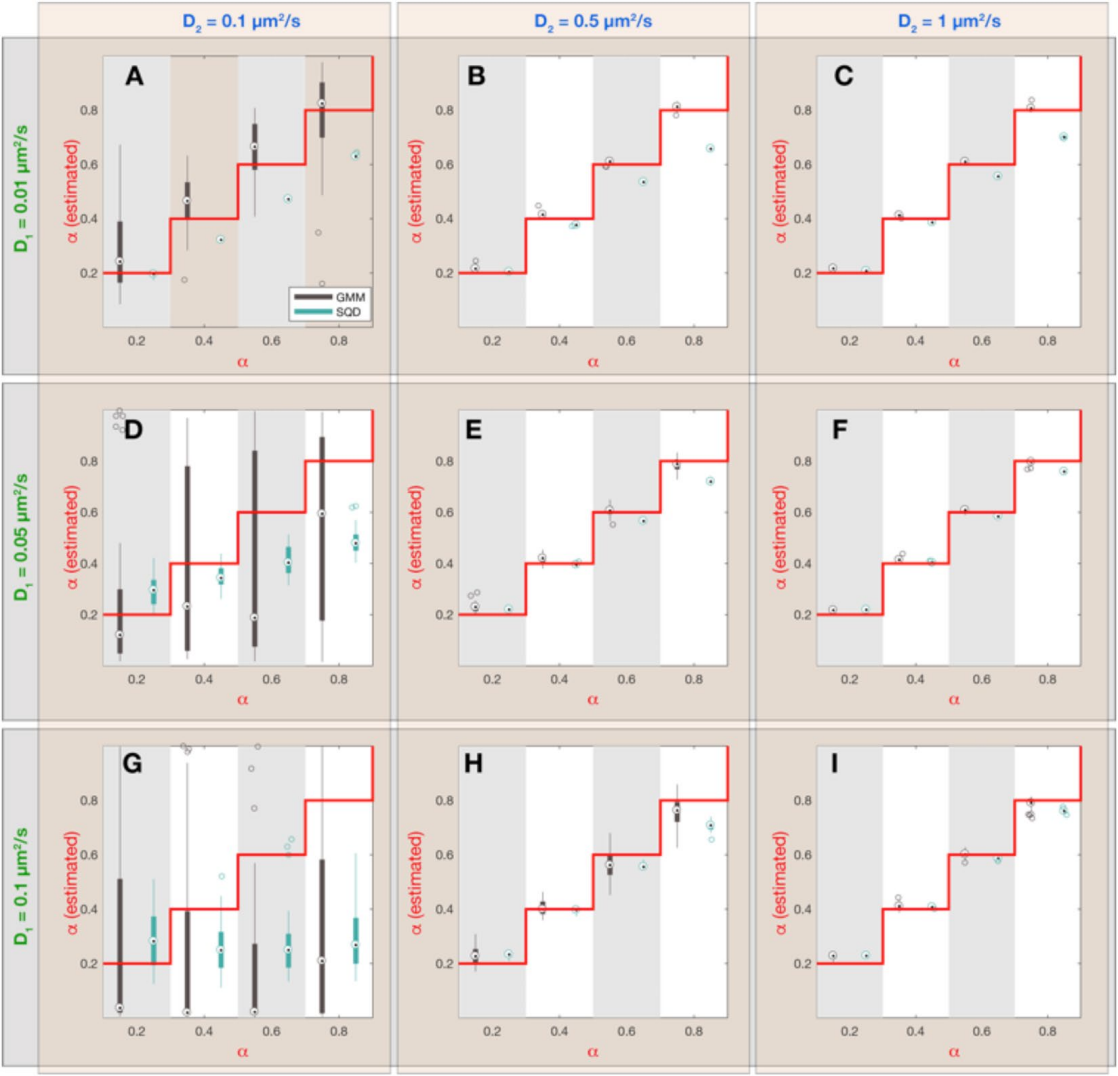
Dependence of estimated fraction ${\alpha }$ (slow subpopulation) on simulation parameters and inference method. Each panel (A–I) corresponds to a combination of simulation parameters ${D}_{1}$ and ${D}_{2}$, as indicated by the green and blue labels outside the plot panels, respectively. Within each panel 4 values of the fraction sizes $\alpha$ were chosen and synthetic simulations were performed on all parameter combinations. For each parameter combination (${D}_{1}$, ${D}_{2}$ and $\alpha$) 30 simulations with 2000 tracks of 20 frames duration (see Table 1 for all simulation parameters) were executed and the resulting data was analyzed with the GMM and SQD method, yielding two different estimates for the value of $\alpha \,$(GMM: black boxplots; SQD: cyan boxplots). On each box, the central mark is the median, the edges of the box are the 25^th^ and 75^th^ percentiles, the whiskers extend to the most extreme data points not considered outliers and the outliers are plotted as individual circles. The red lines indicate the true values for $\alpha$ in the simulation.

For benchmarking the performance of the GMM and SQD methods, we simulated a set of synthetic datasets varying in the diffusion constants ${D}_{1}$ and ${D}_{2}$, as well as in the fraction $\alpha$ of the slow-diffusive subpopulation of molecules (Figs 2–4). Each dataset consists of 30 simulation runs with 2000 single molecule tracks with a length of 20 frames. The tracks of each synthetic dataset were analyzed via the GMM and SQD methods and the resulting estimated parameters were compared with the true values entering the simulation (Fig. 2 – ${D}_{1}$; Fig. 3 – ${D}_{2}$; Fig. 4 – $\alpha$). Overall, we found that both methods capture the diffusion constants and the fraction sizes of the two subpopulations with reasonable accuracy, provided that the diffusion constants are significantly different from each other and both range in a regime amenable to experimental resolution, as detailed further below.

When comparing both inference methods, it became apparent that the SQD method is more accurate in inferring the diffusion constant of the slower subpopulation (Fig. 2) while the GMM method is more precise in estimating the diffusion constant of the fast subpopulation (Fig. 3). Intuitively, this performance difference is related to the fact that the GMM method only takes into account the displacements between single frames, while the SQD method considers the displacements between multiple (up to 4) frames simultaneously. Accordingly, if the displacements are small (as for the case of the slowly diffusing fraction of molecules) the SQD method averages over more displacements and thereby achieves a higher accuracy in estimating the diffusion constant than the GMM method. Note, however, that both methods can only resolve diffusion constants larger than a lower limit determined by the finite localization precision ${{\rm{\sigma }}}_{loc}$ of the experimental setup, as given by ${D}_{loc}=\frac{{{\rm{\sigma }}}_{loc}^{2}}{2{\rm{\Delta }}t}\,$(Fig. 2; *dashed lines*). On the other hand, if displacements are large (as for the case of the rapidly diffusing fraction of molecules) averaging the displacements over multiple frames leads to a slight underestimation of the diffusion constant by the SQD method (Fig. 3), because the longer the observation timescale, the more likely the trajectory is restricted by the confinement to the cell volume, leading to an apparent reduction in the observed displacements. To provide the user with the ability to flexibly adjust the accuracy of the SQD method to either slow or fast diffusive molecules we further implemented the option to choose between fits considering $t=1{\rm{\Delta }}t,\,2{\rm{\Delta }}t,\,3{\rm{\Delta }}t,4{\rm{\Delta }}t$ time frames or all of them simultaneously. Thus, in order to obtain high accuracy in the estimation of low diffusion constants the user should choose to take all time frames into account, whereas for accuracy in the estimation of high diffusion constants the best choice will be $t=1{\rm{\Delta }}t$. Under the latter conditions the GMM and SQD methods are in fact equivalent and yield identical results (Figs S10–S12). Comparing the estimated fraction sizes $\alpha$ shows that for most parameter combinations both methods show a comparable accuracy, with estimated fraction sizes deviating from the true fraction sizes by less than 10% (Fig. 4). Only if the diffusion constant of the slow and the rapid subpopulation are (almost) identical (Fig. 4D,G; ${D}_{1}=0.05,0.1\,\mu {m}^{2}/s;{D}_{2}=0.1\,\mu {m}^{2}/s$), the variance in the estimated values of $\alpha$ increases, indicating that more than a 2-fold difference in diffusion constants is required to reliably infer the fraction sizes of two subpopulations.

### Transition state regulator AbrB shows highly dynamic binding throughout the *Bacillus subtilis* nucleoids, and forms one or two subcellular clusters on the chromosomes

Next, we applied the SMTracker software to analyze experimentally aquired single molecule tracking data by focussing on the subcellular dynamics of the transcription factor AbrB in the Gram-positive model organism *Bacillus subtilis*. AbrB has long been known as a global regulator of genes whose expression is de-repressed as cells transition from exponential into stationary growth. Due to its structural similarity to small nucleoid-associated proteins in bacteria (often also called histone-like proteins), it has been speculated that AbrB may also confer a structural role in chromosome organization, in addition to its specific role in gene regulation. To investigate this point from a cell biological perspective, we generated a C-terminal fusion of YFP to AbrB in *B*. *subtilis*, which was still able to support biofilm formation (data not shown), indicating that it functions similar to wild type AbrB. The fusion was expressed from the original gene locus as sole source of the protein in the cell, under control of the original promoter, ensuring that wild type levels are expressed.

**Figure 5:**
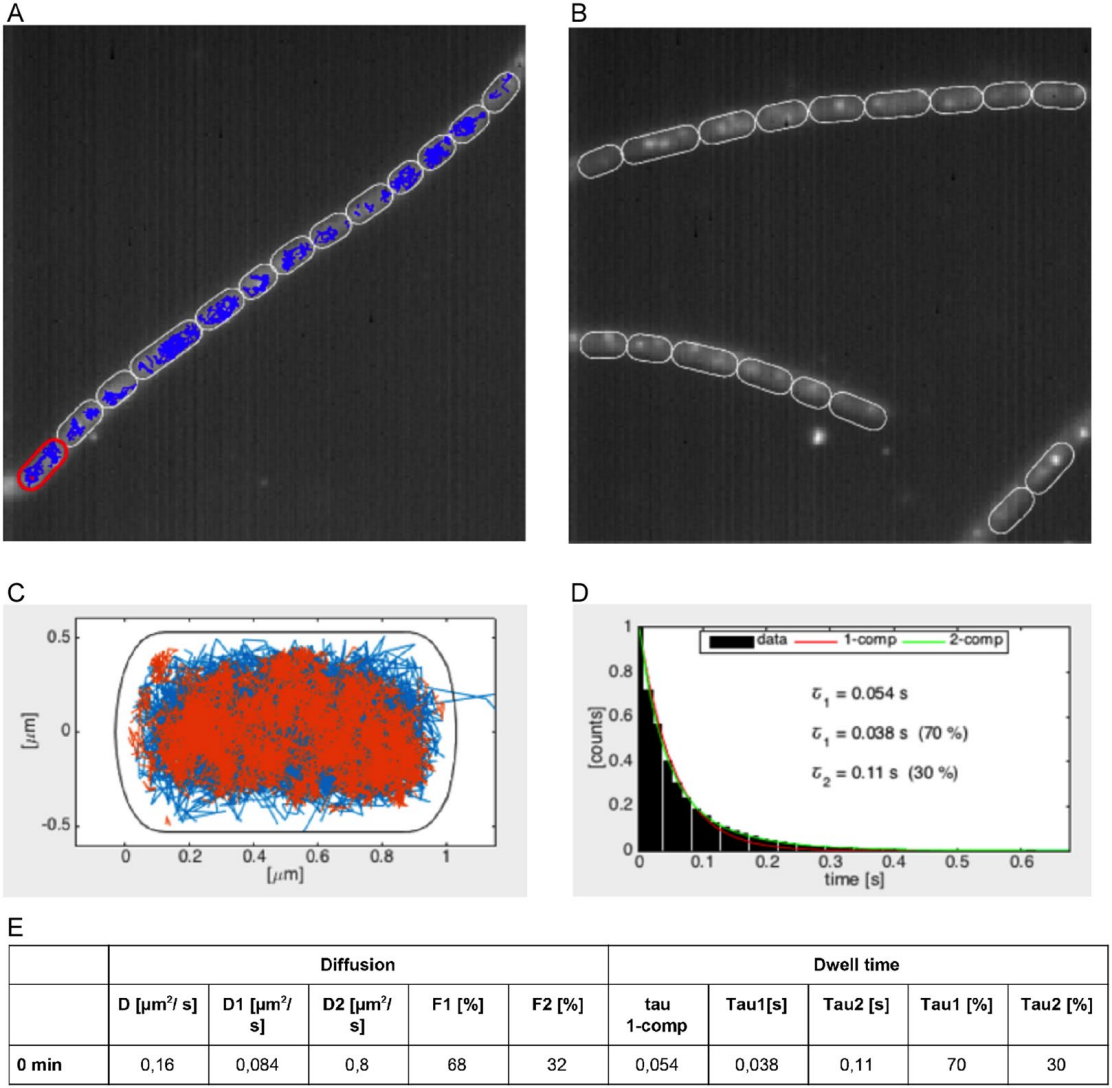
SMT analyses of AbrB-YFP from *Bacillus subtilis*. (**A**) Projection of all frames of a typical stream acquisition overlayed with tracks (in blue) detected by U-track (left panel), (**B**) and a projection of frames without tracks. (**C**) Heat map showing mobile AbrB-YFP tracks in blue, and static tracks in red. (**D**) dwell time determination, (**E**) Diffusion constants and average dwell times of AbrB-YFP.

Using 15 ms stream acquisitions, we obtained 1900 tracks from 66 cells, having an average duration of 180 ms. Using SMTracker, we characterized AbrB dynamics using above-mentioned features. Figure 5A shows a projection of all fluorescence intensities obtained in a chain of cells over time, revealing that AbrB is mainly restricted to the centrally located chromosome(s). In Fig. 5C, the positions of AbrB-YFP tracks in a slow/static state (*red*) and mobile state (*blue*) are shown within a standardized cell of 3 × 1 µm, revealing that AbrB binds throughout the entire nucleoid, and thus throughout the chromosome. This is in agreement with its more than 600 binding sites distributed across the genome^21^. GMM analyses show that 68% of the time AbrB molecules move with a diffusion constant of 0.084 µm^2^/s, whereas in 32% of the time they move with a diffusion constant of 0.8 µm^2^/s (Fig. 5E). Interestingly, the diffusion constant for the fast subpopulation of AbrB (total mass of 156 kDa including YFP) is very similar to the diffusion constant of 0.53 µm^2^/s for the freely diffusive fraction of the Smc dimer^33^ (total mass of 330 kDa including YFPs), and 0.51 µm^2^/s for the freely diffusive fraction of the DnaA dimer^9^ (total mass of 156 kDa including YFPs), suggesting that this corresponds to the freely diffusive state of AbrB. In contrast, the previously reported diffusion constant for the DNA-bound state of Spo0J (0.02 µm^2^/s)^9^ is about 4-fold lower than that for the static subpopulation of AbrB (0.084 µm^2^/s), suggesting that this value might be the result of a mixture between two diffusive states, for instance by AbrB frequently interacting with DNA in a non-specific manner, in search of its binding sites. Indeed, a more fine-grained analysis using the 3-state SQD approach support the presence of 3 populations: D = 0.019/0.1/ 0.81 µm^2^/s; F = 35/40/25%, suggesting that 35% of AbrB molecules are bound to their target DNA sites, while 40% move along DNA in a constrained manner, and 25% freely diffuse as tetramers.

**Figure 6:**
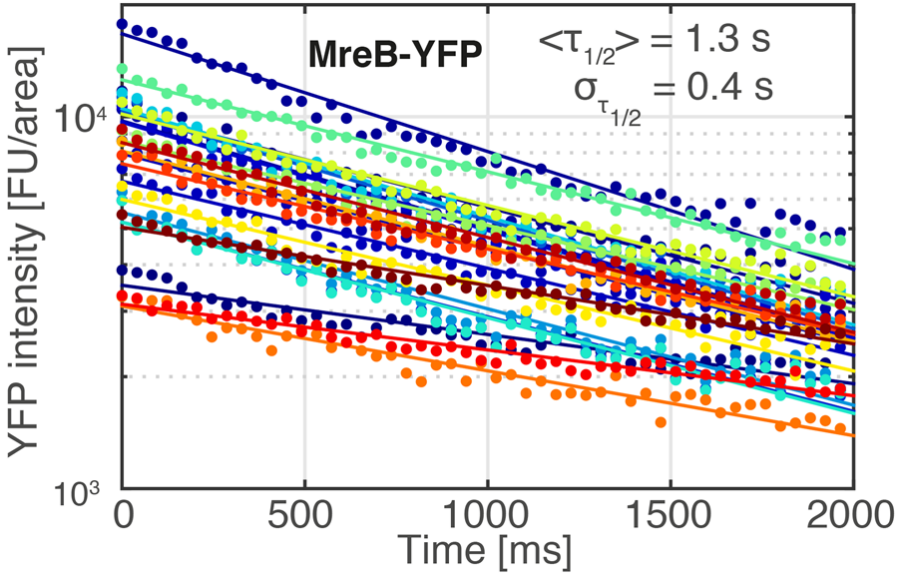
Bleaching of YFP-MreB in single *B*. *subtilis* cells. Image acquisition parameters were identical to those used in Fig. 5. Exponential fits (solid curves) to the bleaching curves of 23 single cells (dots) revealed an average bleaching half-life time of <*τ*_1/2_> = 1.3 s with a standard deviation of σ_1/2_ = 0.4 s.

Figure 5D shows SMT-derived dwell time determination of AbrB-YFP, revealing two distinct fractions – one with 38 ms, and the other with 110 ms. The latter fraction likely represents the DNA-bound AbrB fraction, and because its dwell time is shorter than the average life time of AbrB tracks, it reveals that binding of AbrB is very dynamic *in vivo*. It should be noted that actual dwell time *in vivo* will be somewhat longer, because of YFP bleaching due to the experimental setup. To test if bleaching strongly influences dwell time determination, we used a YFP-MreB fusion and determined bleaching kinetics of static MreB molecules (Fig. 6), which are bound within filaments that run underneath the cell membrane mostly perpendicular to the long axis of cells^34^, and play an important role in the maintenance of cell morphology. Average half-lifetime of YFP molecules was 1.4 s, much longer than the estimated dwell time of AbrB-YFP, showing that YFP bleaching does not strongly affect dwell time determination of AbrB. Interestingly, an average dwell time of 110 ms was found for 30% of AbrB-YFP molecules (Fig. 5E), in good agreement with the 35% of static AbrB molecules as deduced from SQD analyses. Thus, AbrB binding sites are occupied by individual AbrB molecules for only short times (LacI repressor has average DNA binding times of few minutes^35^), revealing high molecule turnover on the chromosome.

A further interesting finding is shown by overlaying all tracks obtained by SMT acquisition, which reveals the position of static tracks. Figure 5B shows that AbrB-YFP forms static foci in cells, either one or two, on the nucleoids. Although we can presently not determine if these sites correspond to any specific site on the chromosome (e.g. origin regions), and if they confer an important function, it is interesting to note that this is a parallel to global trabscriptional silencer H-NS in *E*. *coli*, an important nucleoid-associated protein with many hundreds of binding sites. In contrast to other nucleoid-associated proteins such as HU, Fis and IHF, which are scatterd throughout the genome, H-NS forms one or two subcellular clusters on the nucleoids that are important for nucleoid shaping^36^. It is therefore possible that AbrB confers a similar structural function in *B*. *subtilis*.

## Conclusions

SMTracker software provides a user-friendly interface for comprehensive visualization and analysis of SMT data that can be readily printed in publication-quality figures in an effortless and automated way. It provides an intuitive pipeline to compare the diffusion of molecules under varying conditions in terms of fraction size, spatial distribution and binding times. Our software was benchmarked on a computer-simulated set of tracking data, exposing the advantages of individual SMT analyses under different experimental conditions, and thereby highlighting the usefulness of combining multiple approaches of SMT data analysis in the SMTracker suite. Using this pipeline, we show that the genome-wide repressor AbrB in *B*. *subtilis* shows three fractions of different mobility, with one third of the molecules being bound to DNA throughout the genome, about one third sliding along DNA in search of binding sites, and another third freely diffusing. As AbrB has very short dwell times on DNA, our data suggest that there is a high exchange between molecules being bound and diffusing along DNA, and between the latter fraction and freely diffusing molecules. Although SMTracker was used for SMT in rod-shaped bacterial cells, SMTracker can be applied to SMT experiments in cells with different morphologies even up to eukaryotic cells (e.g. *Schizosaccharomyces pombe*). We expect that SMTracker will enable non-specialized scientists to answer advanced questions in (bacterial) cell biology without the need to deeply engage in programming and the underlying mathematical analyses. Finally, SMTracker allows data export to other tools such as SMMtrack^9^ and the vbSPT software^37^ (see suppl. material), which analyzes SMT data on the basis of a Hidden Markov model to extract diffusion coefficients and transition rates between diffusive states.

## Materials and Methods

### Bacterial strains and growth conditions

AbrB was fused to YFP by amplification of 500 bp from the 3′ end of the *abrB* gene (excluding the stop codon) and by Gibson assembly into pSG1164^38^. *B*. *subtilis* strain PY79 was transformed with the resulting plasmid, such that a fusion of the *abrB* gene with *yfp* at its C-terminus was generated at the original gene locus, which was under control of the *abrB* promoter. As *abrB* is monocistronic, no downstream gene was affected. For microscopy, cells were grown in S7_50_ minimal medium at room temperature until mid-exponential growth, and were imaged under laid by agarose containing growth medium.

### Single molecule tracking of AbrB

Imaging was performed with a Nikon Ti-E microscope configured with a high numerical aperture objective (CFI Apochromat TIRF 100XC Oil, NA 1.49), an EM-CCD camera (ImagEM X2, Hamamatsu) and an filter set for imaging YFP molecules (YFP HC Filterset; BrightLine 500/24, Beamsplitter 520 and BrightLine 542/27). Specimen were continuously illuminated with the central part of an expanded laser beam (TOPTICA Beam Smart, 515 nm, max. power 100 mW) with an intensity of 160–300 W/cm^2^ and streams were recorded at a frame rate of ~67 Hz using VisiView (Visitron Systems).

### Software implementation

SMTracker was developed for Windows and Mac OS X running MATLAB R2014b and later. The graphical user interface (GUI) of SMTracker relies on the GUI layout Toolbox (Ben Tordoff and David Sampson, Consulting Services group at MathWorks), which is freely available at https://www.mathworks.com/matlabcentral/fileexchange/47982-gui-layout-toolbox. In addition, SMTracker requires the following MATLAB toolboxes: Curve Fitting, Statistics and Machine Learning, Optimization, Image Processing and Parallel Computing. SMTracker is available under GPLv3 license at https://sourceforge.net/projects/singlemoleculetracker, where updated versions can be downloaded for direct use as well as for further extension and modification by experienced developers. The authors request acknowledgment of the use of SMTracker in published works.

## Electronic supplementary material

Supplementary Dataset 1

## Data Availability

All data are availabile within the manuscript.
