# Supplementary material for: SMTracker: a tool for quantitative analysis, exploration and visualization of single-molecule tracking data reveals highly dynamic binding of B. subtilis global repressor AbrB throughout the genome
Source: Sci Rep. 2018 Oct 24;8:15747. doi: 10.1038/s41598-018-33842-9 (PMC6200787; doi:10.1038/s41598-018-33842-9)
Supplement: Supplementary file 1 — Supplementary Dataset 1 [file 41598_2018_33842_MOESM1_ESM.pdf]

SMTracker

User Manual

Single Molecule Tracking Analysis

GENERAL INFO 3
REQUIREMENTS 3
LICENSE 3

GETTING STARTED 4
INSTALLATION 4
PROGRAM OVERVIEW 4
DATA STRUCTURE 4
RUNNING THE SOFTWARE 6
IMPORT AND EXPLORE DATA 6
TRACK EXPLORER 10
GAUSSIAN MIXTURE MODEL (GMM) TAB 11
MEAN SQUARE DISPLACEMENT (MSD) TAB 12
SQUARE DISPLACEMENT ANALYSIS (SQD) TAB 14
SPATIAL DISTRIBUTION (SDA) TAB 15

ADDITIONAL INFORMATION 17
RUN VBSPT WITH DATA GENERATED BY SMTRACKER 17
RUN SMMTRACK WITH DATA GENERATED BY SMTRACKER 17
OUTPUT FORMAT OF THE SMTRACKER SOFTWARE 18

REFERENCES 24

General Info

Requirements

For full functionality, SMTracker needs the following requirements:

- Mac OS 10.12.6 or higher
- Windows 7 or higher
- MATLAB 2014b or higher
- Official Toolboxes installed in MATLAB:
    - Statistics and Machine LearningToolbox
    - Curve Fitting Toolbox
    - Image Processing Toolbox
    - Optimization Toolbox
    - Parallel Computing Toolbox
- Custom Toolboxes installed in MATLAB:
    - GUI Layout Toolbox by David Sampson

Recommended software packages:

- U-track: http://www.utsouthwestern.edu/labs/danuser/
- TrackMate: https://imagej.net/TrackMate
- MicrobeTracker microbetracker.org
- Oufti: http://oufti.org/

License

This program is free software: you can redistribute it and/or modify it under the terms of the GNU General Public License as published by the Free Software Foundation, either version 3 of the License, or (at your option) any later version.

This program is distributed in the hope that it will be useful, but WITHOUT ANY WARRANTY; without even the implied warranty of MERCHANTABILITY or FITNESS FOR A PARTICULAR PURPOSE. See the GNU General Public License for more details.

You should have received a copy of the GNU General Public License along with this program. If not, see http://www.gnu.org/licenses/.

Getting Started

Installation

Unzip the content of the SMTracker package. Start MATLAB and add the extracted folder "SMTracker 1.0" to the search path by either using the dialog box *Set Path → Add with Subfolders* or by using the command line:

```
>> addpath(genpath(uigetdir))
>> savepath
```

Please make sure to install the GUI Layout Toolbox v2.2.1 and add u-track/Trackmate to the path before running SMTracker.

Program overview

The SMTracker software is designed to analyse single-molecule tracking data mainly in prokaryotic cells, but it is easily extendable to other organisms. The program offers various ways of analysing single-molecule diffusion in terms of localization, mode of diffusion, identification of various populations in the sample and molecular binding times. SMTracker combines information from tracking data and segmentation data to analyse single-molecule diffusion on a cell-by-cell and track-by-track basis.

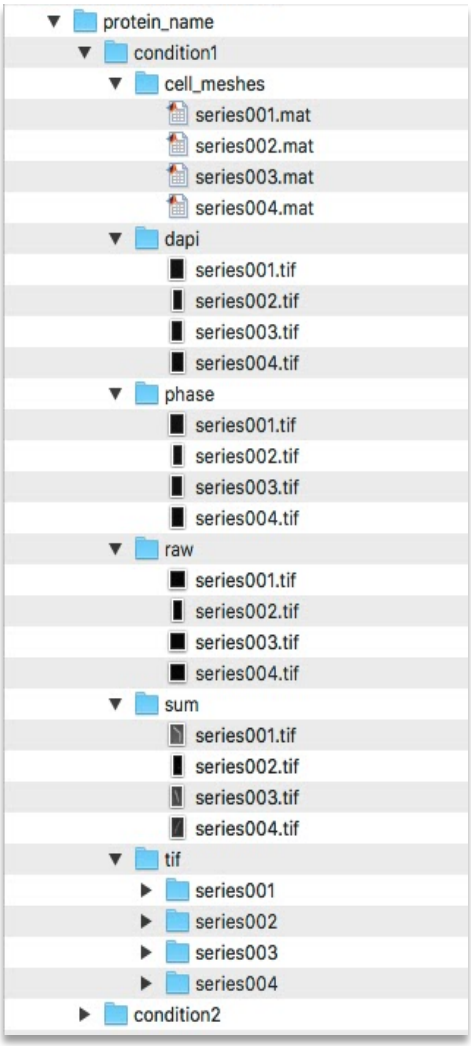

Figure 1 | Folder structure and file nomenclature. Data are organized in a tree-like structure such that a protein can be analyzed by different conditions, e.g. condition1 and condition2. During imaging, pictures were acquired in the DAPI and the phase contrast channel, and afterwards raw movies were processed into "sum" images (temporal average of all frames), tracked ("tif") and cell contours were produced ("cell_meshes").

Data structure

SMTracker handles the analysis of several single molecule fluorescence movies from different treatments or conditions in parallel. To import and match all data of an experiment, it is <u>mandatory</u> that the corresponding files

are named identically. To this end, phase contrast or bright field images, cell mesh files produced by Oufti or MicrobeTracker and folders generated in u-track need to be named in an ascending order and saved in the corresponding folder (**Figure 1**).

**Folder**	**Description**
cell_meshes	Folder must contain standard MATLAB ".mat" files generated in Oufti or MicrobeTracker. The SMTracker software reads the variable "cellList" and imports the variables mesh, model, box and length from each cell in the object "cellData" (see Table M4).
dapi	Folder may optionally contain pictures acquired using any fluorophore as long as the file format is ".tif". User can leave this folder empty to analyse their data.
phase	Folder may optionally contain pictures acquired using any fluorophore as long as the file format is ".tif". User can leave this folder empty to analyse their data.
raw	Folder may optionally contain multidimensional or multi-image ".tif" files, which can be visualized within the SMTracker software and which were used for the tracking by u-track or TrackMate. User can leave this folder empty to analyse their data, but it is not possible to watch the movie in the SMTracker software.
sum	Folder may optionally contain pictures acquired using any fluorophore as long as the file format is ".tif". User can leave this folder empty to analyse their data.
tif	Folder must contain the output folder from u-track. Basically, each folder contains the following files and folders (**Figure 2**):<br>- movieData.mat → Stores movie information such as channels, number of frames, pixel size, time interval<br>- *seriesXXX* → Folder stores each frame of the multi-image ".tif" file as single TIFF image<br>- *seriesXXX.mat* → Same as movieData.mat<br>-TrackingPackage → Stores localization / detection data in folder "GaussianMixtureModels" and single-molecule tracks in the folder "tracks". Each folder contains a standard MATLAB file accounting for its specific information (Channel_1_detection_result.mat and Channel_1_tracking_result.mat)

Table 1 | Description of folder contents, file types and data requirements if u-track is the chosen tracking software.

Please use *seriesXXX* as the base of the filenames. Note that it is recommended to set up the data structure and insert data into this structure before starting the analysis process including tracking, cell contour determination or image processing. If u-track is the chosen software for tracking, please put the raw movie files into the "tif" folder and later move it to the "raw data" folder. Once saved in the folder called "raw", movies can be loaded and played in the SMTracker software. A general description of the individual folders and/or files stored in the data structure can be found in **Table 1**.

If TrackMate is the chosen software for tracking (Tinevez *et al.*, 2016), the user needs to store the output files from TrackMate (appropriately named according to the requirements above) in the condition folder as subfolders “trackmate/tracks”. Please note, SMTracker does not read the TrackMate *.xml* session file, but the tracking file exported in TrackMate using “Export tracks to XML file”. The SMTracker imports the *x*- and *y*- coordinates of the single particles and the frame number where the particle was detected.

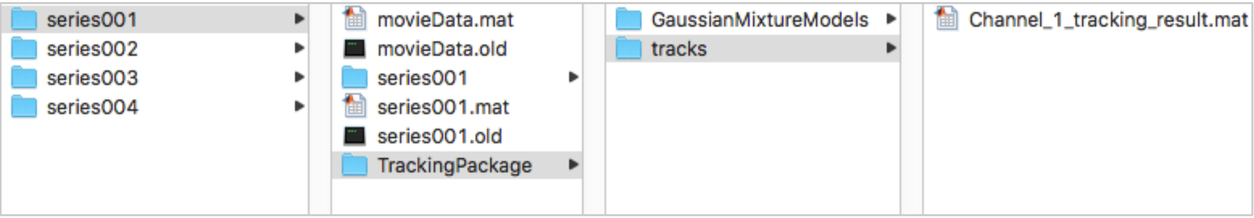

Figure 2 | Folder structure in the “tif” folder generated by the u-track software.

Running the software

To run the SMTracker software, launch its graphical user interface by typing in the Command Window:

```
>> SMTracker
```

Import and explore data

The software opens and shows the IMPORT panel (**Figure 3**), which allows the user to either import data into the software or to load previously analyzed data. To start a new analysis using data from u-track, just click “*Select Folder*”. If the source of data is TrackMate, please first provide the pixel size in nanometers [nm] and the time interval in milliseconds [ms] and then click “*Select Folder*”. A dialog box opens and allows navigating to the folder containing the datasets. Selecting the folder “*protein_name”* (**Figure 1**) start the import of all data contained in the sub-directories “*condition1”, “condition2”, etc*. If successful, the panel will be populated with additional features that provide full software functionality (**Figure 4**), as explained below. To load a dataset that was analyzed and saved before, go to *File → Load, and* select the file *“protein_name.mat”*. If the tracking data produced is with TrackMate, first go to *File → Type of data and* select *TrackMate.* Then, proceed as above.

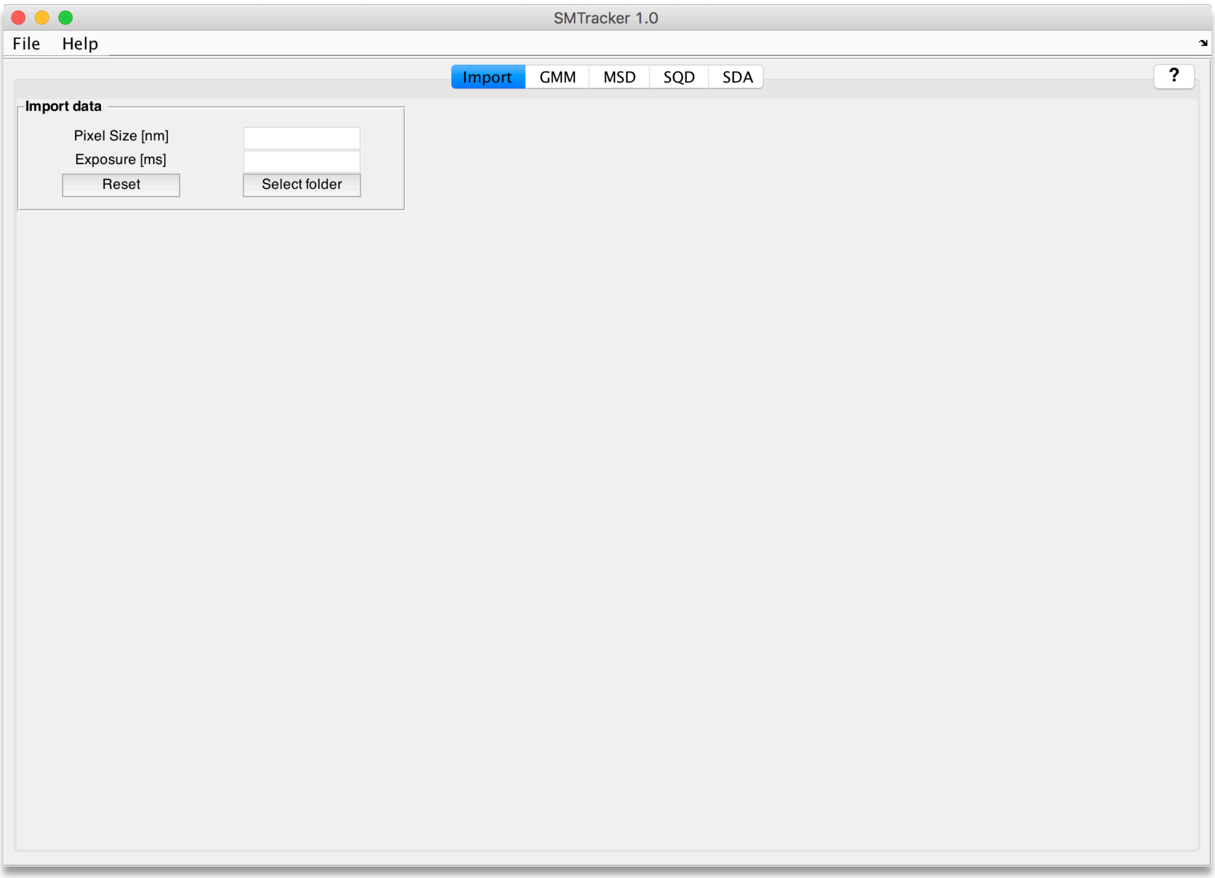

Figure 3 | Data import. Provide pixel size of the microscopy system in nanometers and the time interval between image frames in milliseconds (only for TrackMate datasets). Then click "Select folder" and proceed by selecting the folder containing the conditions.

The IMPORT tab has the following components:

Menu bar:

- *File:* Uses the File menu to load, save, export and import, or select the type of data.
  - *Select Folder:* Loads a dataset.
  - *Load/Save:* Loads/saves a session of SMTracker.
    Type of Data: Selects software source that generated the tracking data. Currently, SMTracker can import data from u-track [default] or TrackMate.
  - *Export:* Data can be exported into two different structures: vbSPT (Perrson *et al.*, 2014) or SMMTrack (Schenk *et al.*, 2017). Please see Additional Information at the end of the document for further instructions.
  - *Import:* Imports residence times calculated in SMMTrack.
  - *Print figures:* Use the print menu to open a dialog box, which allows saving and printing publication-ready high-quality figures (300 dpi) in different formats.

- o *Exit:* Exit SMTracker
- Help: User Manual and Information about the developers

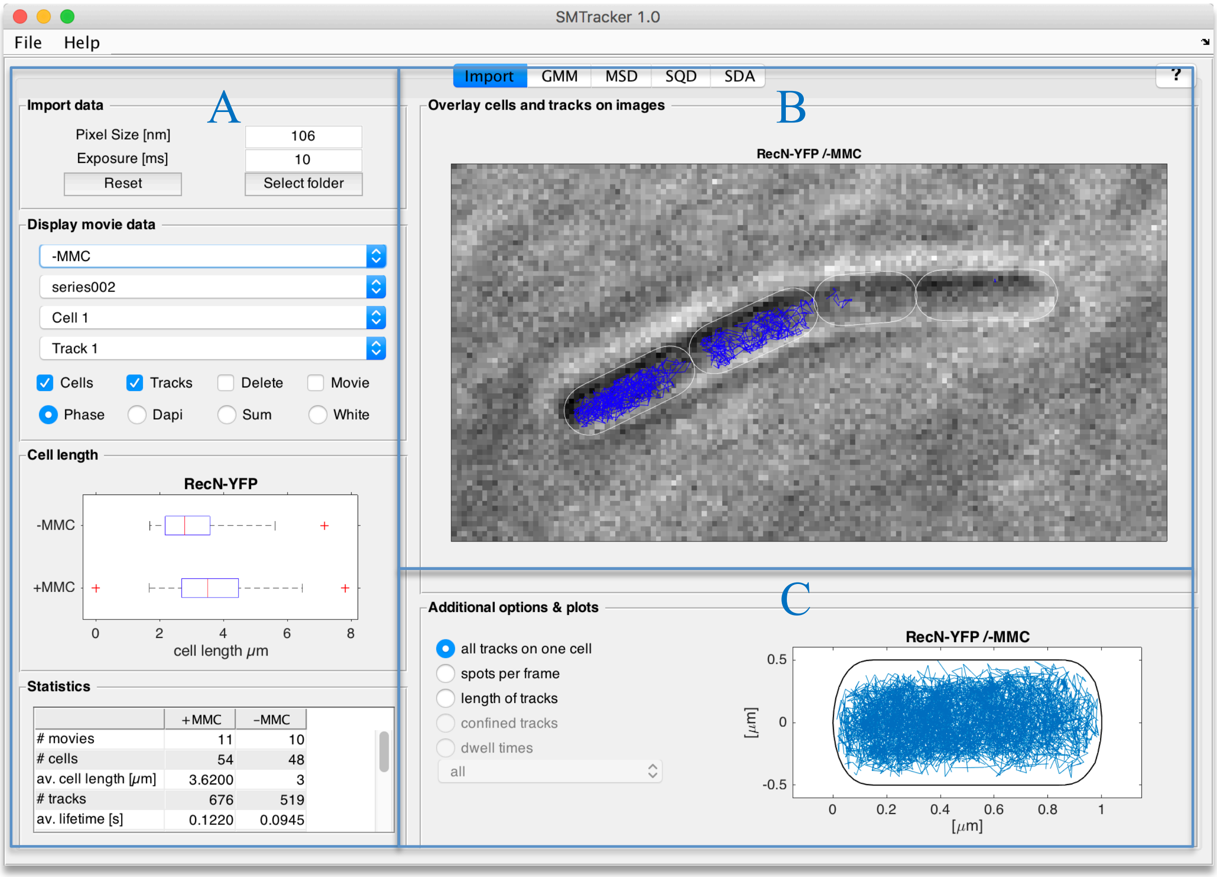

Figure 4 | Import panel after data import. (A) Import data panel, navigation selectors and general statistical data of the sample. (B) Pictures from the channel selected are displayed in this panel, plus cell contours and tracks, according to the selectors in Display movie data panel. (C) Some additional plot such as overlay of normalized tracks; histograms of lengths of tracks and additional plots are displayed in the Additional options & plots panel.

Panels:

- **Import data:** Click "*Select Folder*" to search and load the protein folder. Click on *"Reset"* button to clear the window.
- **Display movie data:** Uses the popup list to navigate through conditions, movies, cells and tracks. Radio buttons ("*Cells*" and "*Tracks*") and checkboxes ("*Phase*", "*DAPI*", "*Sum*" and "*White*") can be used to show different channels as background in the panel "Overlay cells and track on images", and overly them with cell contours or recorded tracks. If a track needs to be removed, it can be deleted using the checkbox "*Delete*". It removes the selected track from the internal data structure of the SMTracker software but not from the original tracking

file. The checkbox "*Movie*" opens a new figure and plays the movie of single molecule tracks overlaid with the corresponding cell contours.

- **Cell length:** Boxplot of the cell length measurements for each condition.
- **Statistics:** Statistical and quantitative information about the datasets. Further details of the fields are given in **Table 2**.
- **Overlay cells and tracks on images:** Shows the channel selected in panel "*Display movie data*" as background, possibly overlaid with cell contours (in white) and tracks (in blue). Here, individual tracks can be selected by clicking directly on them, which opens the "*Track explorer*" interface, providing extended information about each track.
- **Additional options & plots:** This panel provides additional information about the movies from the selected condition such as:
    - *All tracks on one cell:* Plots all tracks from one condition in a standardized cell.
    - *Spots per frame:* Mean number of spots detected per frame for all movies.
    - *Length of tracks:* Histogram of the length (in # of frames) of the tracks.
    - [*] *Dwell times:* The duration a particle resides inside a defined radius (see Supplementary Text for definition).
    - [*] *Confined tracks:* Plots of tracks classified into confined (red) and not confined (blue).

#movies	Number of imported movies
#cells	Number of cells that contain tracks
av. cell length (μm)	Mean cell length of cells that contain tracks
#tracks	Number of tracks inside cells
av. lifetime [s]	Mean lifetime of tracks
#static tracks	Number of static tracks determined in "*confined tracks*"
#mobile tracks	Number of mobile tracks determined in "*confined tracks*"
τ (1-comp.) [s]	Dwell time for a one-component fit
τ_1 / τ_2 (2-comp.) [s]	Dwell times for a two-components fit
τ_1 / τ_2 [%]	Size of fraction belonging to τ_1 or τ_2

Table 2 | Detailed description of fields in the Import/Statistics table.

[*] To enable this feature, the user needs to calculate previously the double fit in the GMM tab (**Figure 3**).

Track explorer

Once a track or a cell is selected either in the "*Display movie data*" panel or by directly selecting it in the "*Overlay cells and tracks on images*", the "*Track explorer*" interface appears on the right side of the main window (**Figure 5**). The "*Track explorer*" is composed of five panels displaying the following information:

- **Projection of tracks on cells:** The cell containing the selected track is aligned horizontally to display the long axis of the cell parallel to the x-axis. Either the first track in the cell or the selected track is highlighted in a color-coded manner indicating the start of the track in red and the end of the track in blue.
- **Track movement:** Scheme in which the start of the track is set to the origin (0,0) and the movement of tracks is shown relative to the origin in a color-coded manner. The circle indicated by the dashed line is the confinement radius.
- **Distance to origin:** Shows the displacement of the track from the origin over time (black line) and the single frame-to-frame displacement (green line). The dashed line in red indicates the threshold radius used to calculate the dwell times and to classify the track as confined/not confined. The gap accounts for a transient disappearance of the fluorophore during image acquisition that was allowed during the process of tracking.
- **Intensity profile:** Normalized intensity profile of the track (grey area) as a function of time. If possible, the graph also displays the normalized intensity at the sites where the track starts and ends.
- **Track Movie:** A movie showing the selected track together with a cropped version of the original movie and overlaid with the cell contour.

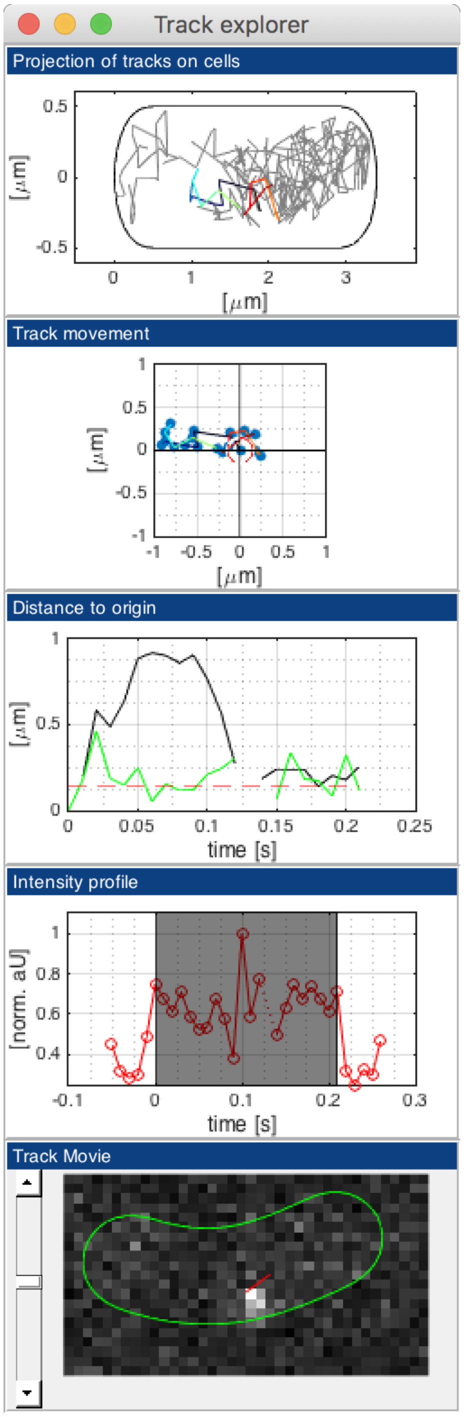

Figure 5 | Track explorer.

Gaussian Mixture Model (GMM) tab

The Gaussian mixture model tab (Figure 6) allows estimating the diffusive properties of two molecular subpopulations across different experimental conditions (details of the GMM method are provided in Roesch et al, 2018). Briefly, the method considers the displacements of molecules in *x*- and *y*-direction between consecutive image frames and fits the resulting histograms with either a single Gaussian probability distribution function (PDF) (single fit) or with a linear combination of up to three Gaussian PDFs (double fit and triple fit), where the mixture parameter $0 \leq \alpha \leq 1$ describes the fraction size of the slowly diffusive subgroup.

The following panels belong to the GMM tab:

- **Axes parameters:** Change of settings for axes and histograms.
- **Data & fits:** Use pop-up menus to select the conditions to be plotted inside the panels on the right-hand side of the main window. The pop-up lists refer to the different folders (conditions) in which your experimental dataset is organized. By default, the software shows the first as "reference" and second one as "comparison" according to the order in the file system. The checkbox "*projected data*" determines whether *x*- and *y*-displacements are given

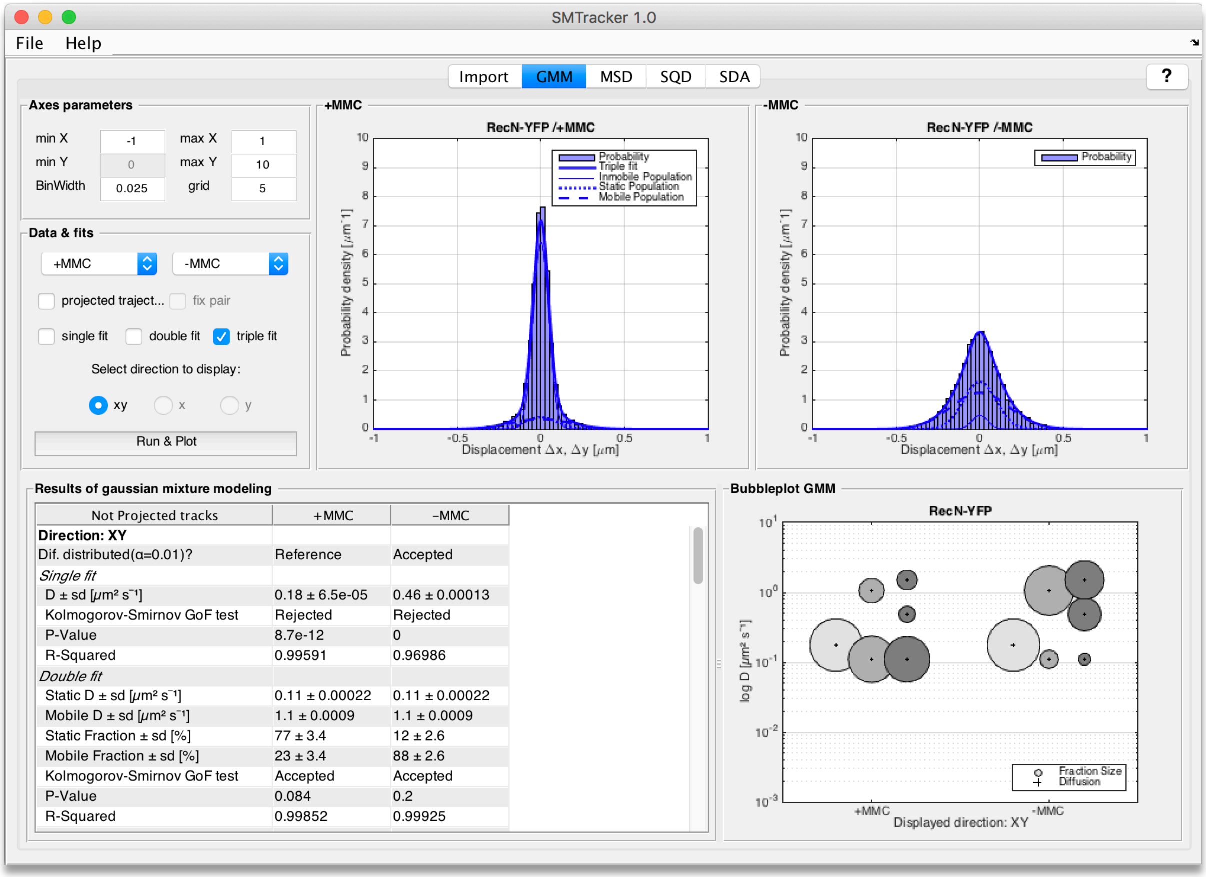

Figure 6 | GMM tab. Histograms (light blue bars) of single-frame displacement data along with triple Gaussian fits (blue lines).

in an image-centric coordinate system ("*projected data*" unchecked) or projected (rotated) to a cell-centric coordinate system ("*projected data*" checked), with the long cell axis paralleling the x-axis. The checkboxes "*single fit*", "*double fit*" and "*triple fit*" run the corresponding Gaussian fits to estimate the underlying parameters. The radio buttons allow the user to select the direction of the displacements: "xy" pools x- and y-displacements together whereas "x" and "y" considers the x- and y-displacements individually. When dealing with more than 2 conditions, *"fix pair"* checkbox enables the user to exclusively the selected conditions on the pop-up lists are pooled together for the fit procedure.

After choosing the settings, click the "*Plot*" button to proceed and new panels appear:

- **Plot Panels:** Histograms of the frame-to-frame displacements in the selected direction superimposed with the PDFs of the corresponding fits.
- **Results of Gaussian mixture modeling:** Best-fit parameters and results from the goodness of fit, single and double-gaussian distribution of the displacements, and significantly different modeled CDFs hypothesis tests are presented in a table. The number of columns corresponds to the number of conditions in the experimental data.
- **Bubbleplot GMM:** Bubble plots showing the diffusion constants of each diffusive group together with their fraction size, as indicated via the area of the bubbles.

Mean Square Displacement (MSD) tab

The most common approach to describe molecular motion is by the mean square displacement (MSD) analysis, which is recommended if only a single population of diffusive molecules is considered. MSD curves are very useful to identify the type of motion exhibited by a particle.

The following panels belongs to the MSD tab (**Figure 7**):

- **Plot ensemble & time-averaged MSD curves:** Select experimental condition for which MSD analysis shall be performed.
- **Parameters to calculate time-averaged MSD:** First, the user can choose if the MSD calculation takes into consideration the entire length of every trajectory (*all* τ) or if it only uses tracks that span a minimum number of time-frames (*specific* τ). Additionally, the user can choose the number n of time lags ($\tau = n\Delta t$) to perform the linear weighted fit. The slope of the MSD curve vs. time-lag curve is proportional to the diffusion coefficient of the proteins.
- **Choose direction:** The MSD can be calculated using different set of data. The radio buttons allow the user to select the source of the displacements: "xy" for a 2D displacement of the localizations between two consecutive frames, "x" and "y" for a displacement along each coordinate axis.
- **Axis scaling:** Sets axis limits.

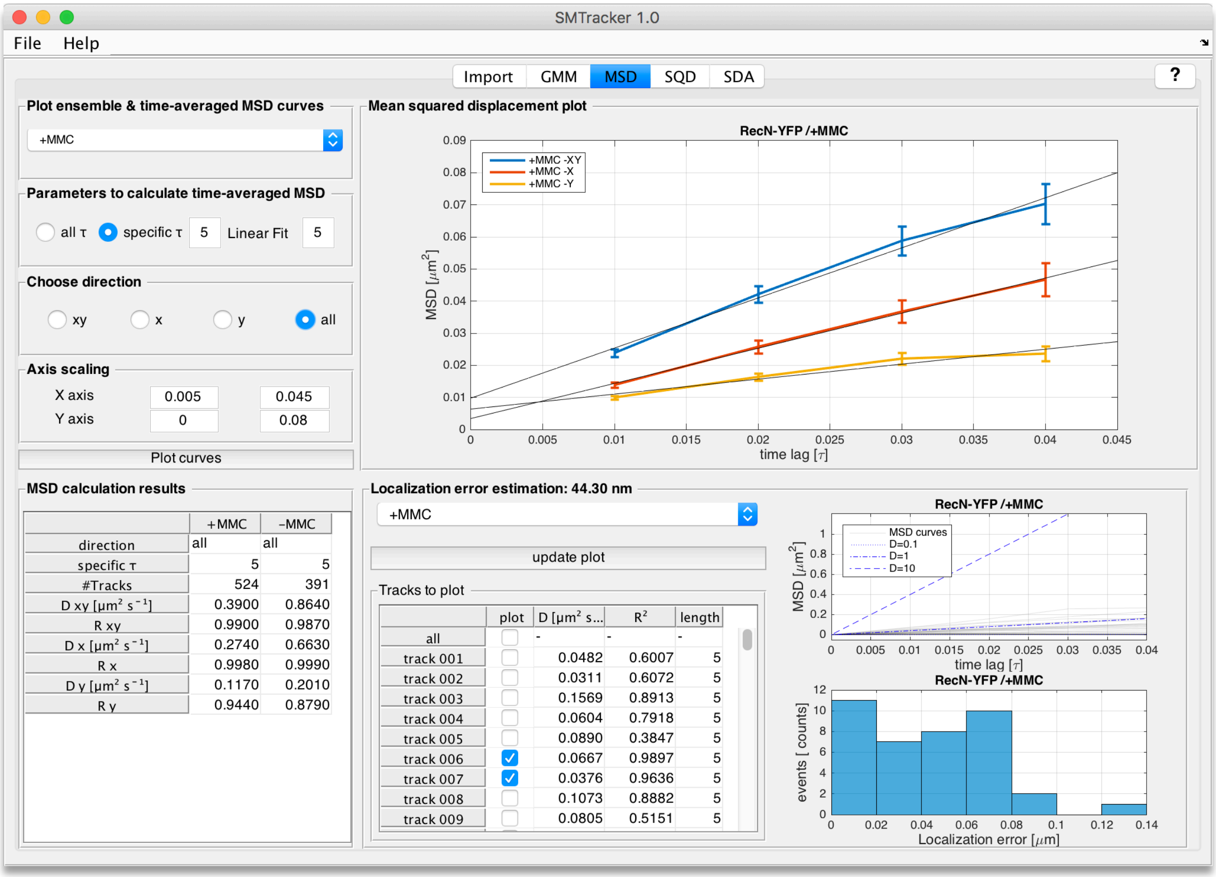

Figure 7 | MSD tab.

After clicking the button "*Plot Curves*", new panels appear inside the MSD tab:

- **MSD calculation results:** Table showing the results from the MSD analysis.
- **MSD Plot:** Following the input parameters, weighted linear fits are shown along with the corresponding data points and the standard deviation. Note that at larger time-lags a lower number of data points contributes to the determination of the MSD values, leading to a natural increase in the error bars.
- **Localization error estimation:** Individual MSD calculations for each track and each time lag are shown inside this panel. In order to estimate the localization error of the sample the user can mark specific tracks. The localization error is calculated using the intersection point of the ordinate axis and the linear fit. Based on the goodness of the linear fit and the confinement of the track, the software suggests tracks from which the localization error can be calculated. All y-intersections are plotted in the histogram and the localization error is printed in the command window.

Square displacement analysis (SQD) tab

The mean squared displacement analysis provides a measurement of the population dynamics, which does not account for heterogeneous movement of single particles or molecules. Besides the introduced GMM method, the SMTracker also offers a second method to assess the diffusive behavior of multiple subpopulations in the sample. The method is based on the cumulative distribution function (CDF) of square displacements r^2, which represents the probability $P(r^2, t)$ that a molecule remains in a circle of radius r in time t. The software allows the user to calculate up to three diffusive groups in a set of trajectories (see Roesch T. et al, 2018 for details).

The following panels belongs to the SQD tab (**Figure 8**):

- **Choose best fit:** SMTracker can estimate the most approximate number of diffusive groups found in the experimental data using the Bayesian information criterion (BIC) (see Supplementary Text). If set to "*Automatically*", SMTracker performs the analysis without taking into account the suggestion of the user. If set to "*Manually*" the user can manually determine the number of diffusive subspecies in the sample.

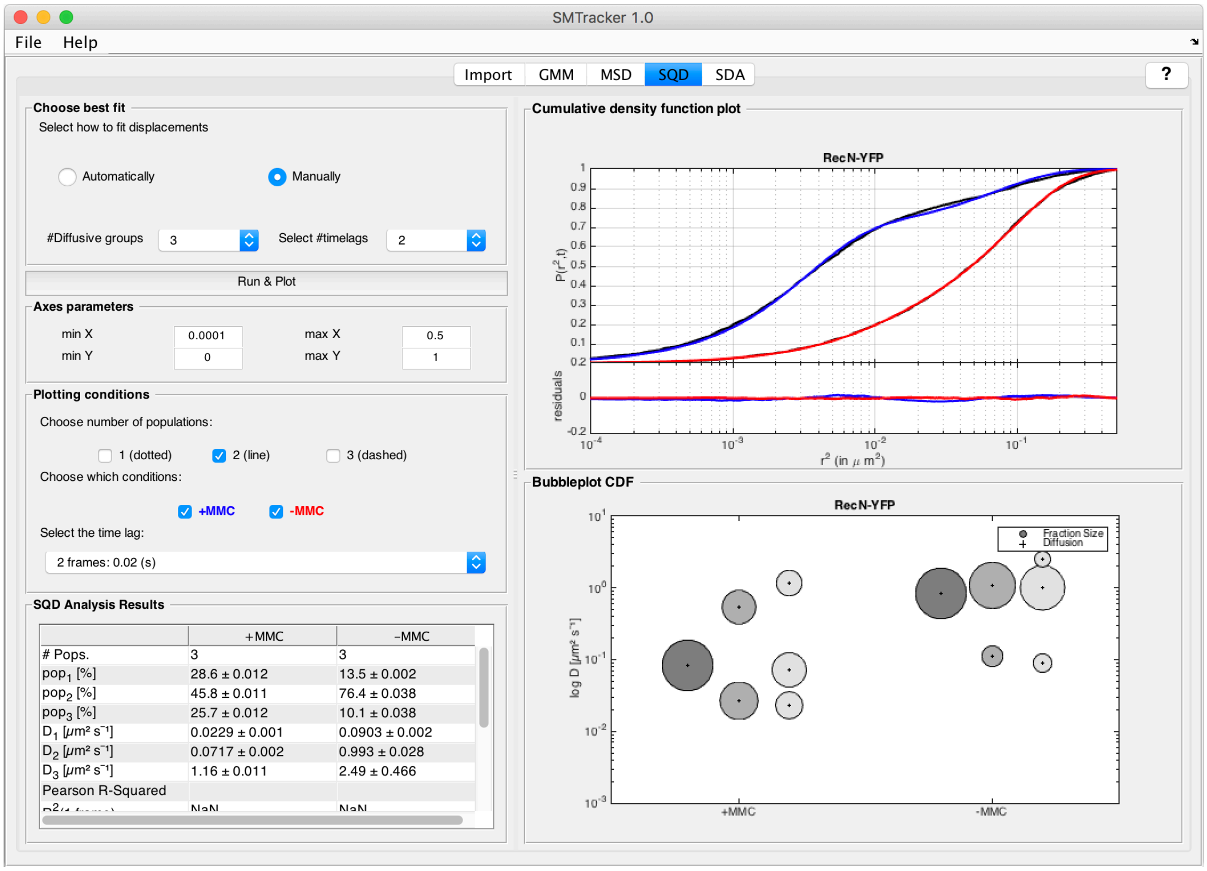

Figure 8 | SQD tab.

After clicking the button "*Calculate fit*", the following result panels appear:

- **Axes parameters:** Settings for the graphs displayed to the right.
- **Plotting conditions:** Click on the conditions and number of diffusive groups checkboxes to select which plot to show and expand the time-lag pop-up to select which one of the four distribution function $P(r^2, t_{lag})$ to plot.
- **CDF Plot:** Empirical cumulative density function of the experimental data superimposed with the fit of $P(r^2, t)$. The lower panel shows the residual differences between model and experiment as a function of r^2.
- **SQD analysis results:** This table shows the results of the analysis, such as the suggested best-fitting model, number of populations selected for the fit, the R-squared for each curve, the diffusion coefficient for both diffusive groups, their fraction size and the result of the difference between calculated CDFs hypothesis test.
- **Bubbleplot CDF:** The plot shows the fraction size, which is proportional to the area of each bubble at the corresponding diffusion coefficients.

Spatial distribution (SDA) tab

To assess the subcellular distribution of molecules, all tracks of each condition are summarized in a heat map and a spatial distributions histogram in the spatial distribution tab (**Figure 9**). To this end all tracks are projected onto a unit cell of 1 x 1 µm.

- **Select conditions:** Choose condition and type of view. Additionally, the user can choose to visualize the heat maps in 2D or 3D or change the number of bins once the plots are shown.

After selecting a condition and cell size category, the following panels appear:

- **Heat map of trajectories:** Spatial localization of particles according to the trajectories info.
- **Heat map with mirroring:** Same as above, but with axes mirroring. This representation takes into account the symmetry with respect to the transversal and longitudinal axis of the cell.
- **Normalized x/y axis distribution:** Normalized histograms of particle positions projected onto the *x*- and *y*-axis. For fine-tuning the histogram representations, the number of bins can be increased or decreased using the corresponding buttons.

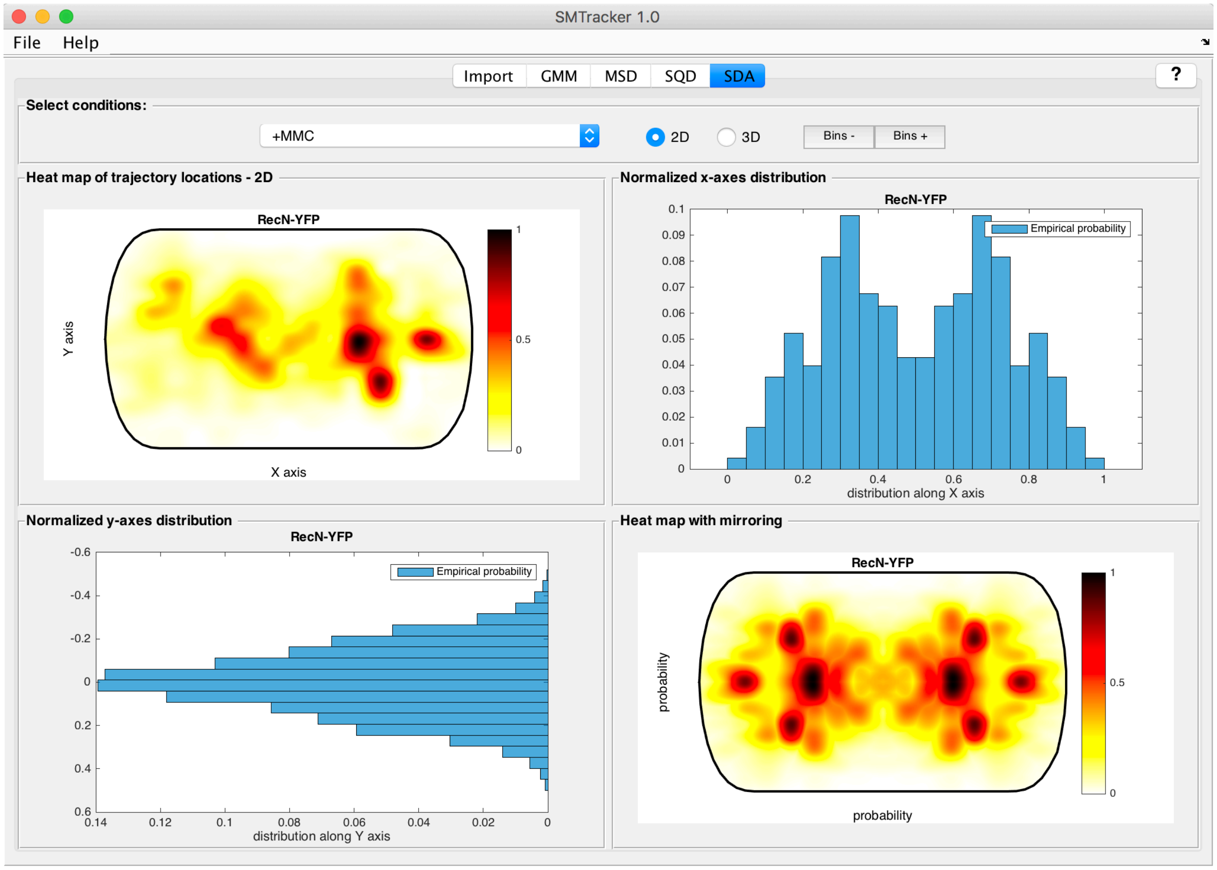

Figure 9 | SDA Tab.

After choosing the export format (SMMTrack or vbSPT), SMTracker saves the *x*- and *y*-coordinates of the trajectories either in a ".csv" or ".mat" format. Files are stored in the folder of the protein under investigation (e.g. "protein_name" in **Figure 1**).

Additional Information

Run vbSPT with data generated by SMTracker

Note: Only use letters and numbers in filenames. Sometimes, we observed that the vbSPT software (Persson *et al.* 2013) produces inhomogeneous results due to outlier data in the trajectories. To remove outliers showing a deviation of more than 10 times the standard deviation of the frame-to-frame displacement, run

```
>> remOutlier
```

and then start the vbSPT user interface:

```
>> vbSPTgui
```

In the “Settings” section enter the following information:

- Choose one of the “.mat” files under “Input data”, which were exported by the SMTracker
- Click “Output data” to define the location where results are stored
- Choose number of Bootstraps, runs, hidden states and minimal trajectory length

In the “Parameters” section enter the following information:

- Set “Units of length” to nm and “Dimensionality” to 2.
- Add the “Timestep” in [s].
- Set the initial range of the diffusion coefficient & the dwell time.
- Click on “Generate ID”

Finally click ”Save” at the bottom of the window to save the *runinput* file (“`runinput_day_month_year.m`”), which stores all input data and the results. Start the analysis by clicking on “Run”. After finishing the analysis to see the results type

```
>> VB3_getResult('runinput_day_month_year.m')′
```

Run SMMTrack with data generated by SMTracker

SMMTrack (https://github.com/SMMTrack; Schenk et al., 2017) runs on most Windows distributions, but to run it on a macOS distribution, download and install Wine or WineBottler (http://winebottler.kronenberg.org/). After installation, create a folder “SMMTrack” where you

store the executable file “SMMTrack.exe” and where you have to move the “.csv” files exported from the SMTracker. Start SMMTrack, go to menu “File” and choose item “read tracks .csv” to load all files in the folder. Note, sometimes “.csv” files need to be first loaded into Excel before they are accepted by the SMMTrack software.

Output format of the SMTracker software

Each SMTracker session can be saved for later analysis. The SMtracker saves three different structures (data, cellstats and params), which will be further explained in the next tables. The field ‘cellData’ in the ‘data’ structure contains all relevant tracking data and is hierarchically ordered in cell arrays. The first array corresponds to the conditions of the experiment, the second layer gives access to all the movies of a condition, and the next layer gives access to the individual biological cells detected for the movie. If the user wants to enter the information stored for the first biological cell in the first movie of the first condition, the user needs to type:

```
>> data.cellData{1}{1}{1,2}
ans =
mesh: [26x4 single]
model: [59x2 single]
box: [1 145 46 61]
length: 23.5529
tracks: {45x1 cell}
projTracks: {45x1 cell}
projCells: [59x2 double]
```

See Table **6** for further explanation of the fields stored in the “cellData” object.

**Field**	**Description**
`protein`	Name of protein folder
`proteinPath`	Path to protein folder
`conditions`	Conditions stored in the protein folder
`movie_names`	Movies names according to movies stored in the subfolder ‘tif’ of the condition folder(s)

`cellMesh_names`	Cell mesh names according to cell meshes stored in the subfolder 'cell_meshes' of the condition folder(s)
`cellList`	Raw cell mesh data from Oufti or MicrobeTracker
`tracks_raw`	Raw tracks read in from the U-track data (tracksFinal)
`movieData`	Raw movieData files read in from the U-track data
`spots`	Raw detection data from U-track (detectionParam, movieInfo, localMaxima and exceptions)
`cellData`	Cell array containing all relevant tracking information
`time_intervall`	Camera integration time (in [s])
`pixel_size`	Size of pixels (in [nm])
`tracks`	Tracks passed over to MSD analysis
`dwell_times`	Stores imported dwell times calculated in SMMtrack
`gmm`	Stores statistics table in GMM panel
`msd`	Stores MSD data according to the direction (xy, x and y)
`sqd`	Struct that stores squared displacement analysis data
`displacement`	Frame-to-frame displacements in xy, x and y direction
`stack`	Imaging data stored in a 3D matrix

tracking_software	'U-track' or 'TrackMate' (user selects format before data import)
f	Stores data for movie plotting/drawing
dim	Dimension
time_scale	[s]
space_scale	[µm]
microbetracker_folder	Name of folder = 'cell_meshes'
microbetracker_var	Variables to load from cell mesh data: {'cellList' 'p'}
phase_folder	Folder name = 'phase'
u_track_folder	Folder name = 'tif'
tracking_folder	Folder name = 'TrackingPackage/tracks'
track_file	File name = 'Channel_1_tracking_result.mat'
spotDetection_folder	Folder names = 'TrackingPackage/GaussianMixtureModels'
spot_file	File name = 'Channel_1_detection_result.mat'
trackmate_folder	Folder name = 'trackmate/tracks'
modelCellContour	Cell contour of a standardized cell

Table 3: Explanation of fields stored in the "data" object.

Table 4: Explanation of fields stored in the "cellstats" structure.

Field	Description
cellLength	Fast access to length of cells for each condition
lftTracks	Fast access to length of tracks for each condition
cdata	Stores statistics table in import panel

Field	Description
version	String with SMTracker name and version
scrsz	Size of screen [x y w h]
minTraLe	Minimal track length
dwellFit	Results of dwell times fit {fitresults, gof}
confined	Confined tracks
xlim	x-axis parameters of GMM figures
ylim	y-axis parameters of GMM figures
BinWidth	Bin width for GMM histograms
xgrid	Number of grids for GMM figures
xtheory_fine	x data to plot fits of GMM

p0	Initial fitting parameter for GMM fit
lb	Lower bounds to start GMM fit
ub	Upper bounds to start GMM fit
triplefit	Structure storing GMM results for triple fits done for each direction
doublefit	Structure storing GMM results for double fits done for each direction
singlefit	Structure storing GMM results for single fits done for each direction
significance_tests	Structure storing results from significance tests
p0_prev	Previous fitting parameter for GMM fit
BIC	Bayesian information criterion to guess best model
view	'U-track'
units	'$\mu m^2 s^{-1}$'
showMessage	Stores parameters for showing messages in the import panel
percentiles	Stores percentiles: [25 75]
npopMAX	Maximum value of SQD diffusive groups = 3
nstepsMAX	Maximum value of SQD time lags = 4
alpha	Significance level = 0.01

heatmatCaxis	Limits for the intensity of localization heat maps.
MSD	Parameters needed to optimize MSD routine
msd_val	Parameters needed to optimize MSD routine
coeff	Parameters needed to optimize MSD routine
prefix	Indicator of data projection = 'proj' or 'nproj '.
ii	Index of selected conditions for GMM analysis
direction	'xy' or 'xy_fix'
sufix	Indicator of fix pair analysis = '_fix' or ' '.
bestModel	String for the best fitted model
bestNpop	Number of components for the best fitted model

Table 5: Explanation of the fields stored in the "params" structure.

**Field**	**Description**
mesh	Dimension of the cell deriving from Oufti or MicrobeTracker
model	Dimension of the cell deriving from Oufti or MicrobeTracker
box	Location of the cell in the image
length	Length of the cell in pixels measured along the long axis

`tracks*`	All tracks detected for the cell
`projTracks`	Projected tracks of the cell
`projCells`	Projected outline of the cell

Table 6: Explanation of the fields stored in the "cellData" object.

*Information stored for each track are: time, x-coordinate, y-coordinate, frame, intensity, standard deviation of x-coordinate, standard deviation of y-coordinate

References

Persson F, Lindén M, Unoson C, Elf J. (2013) Extracting intracellular diffusive states and transition rates from single-molecule tracking data. *Nat Methods* 10:265–9.

Schenk K, Hervas AB, Rösch TC, Eisemann M, Schmitt BA, Dahlke S, et al. (2017) Rapid turnover of DnaA at replication origin regions contributes to initiation control of DNA replication. *PLoS Genet* 13:e1006561.

Schütz GJ, Schindler H, Schmidt T. (1997) Single-molecule microscopy on model membranes reveals anomalous diffusion. *Biophys J* 73:1073–80.

Tinevez, JY.; Perry, N. & Schindelin, J. et al. (2016) TrackMate: An open and extensible platform for single-particle tracking. *Methods* 115: 80-90

Wieser S, Moertelmaier M, Fuertbauer E, Stockinger H, Schütz GJ. (2007) (Un)confined diffusion of CD59 in the plasma membrane determined by high-resolution single molecule microscopy. *Biophys J* 92:3719–28.

Additional Figures

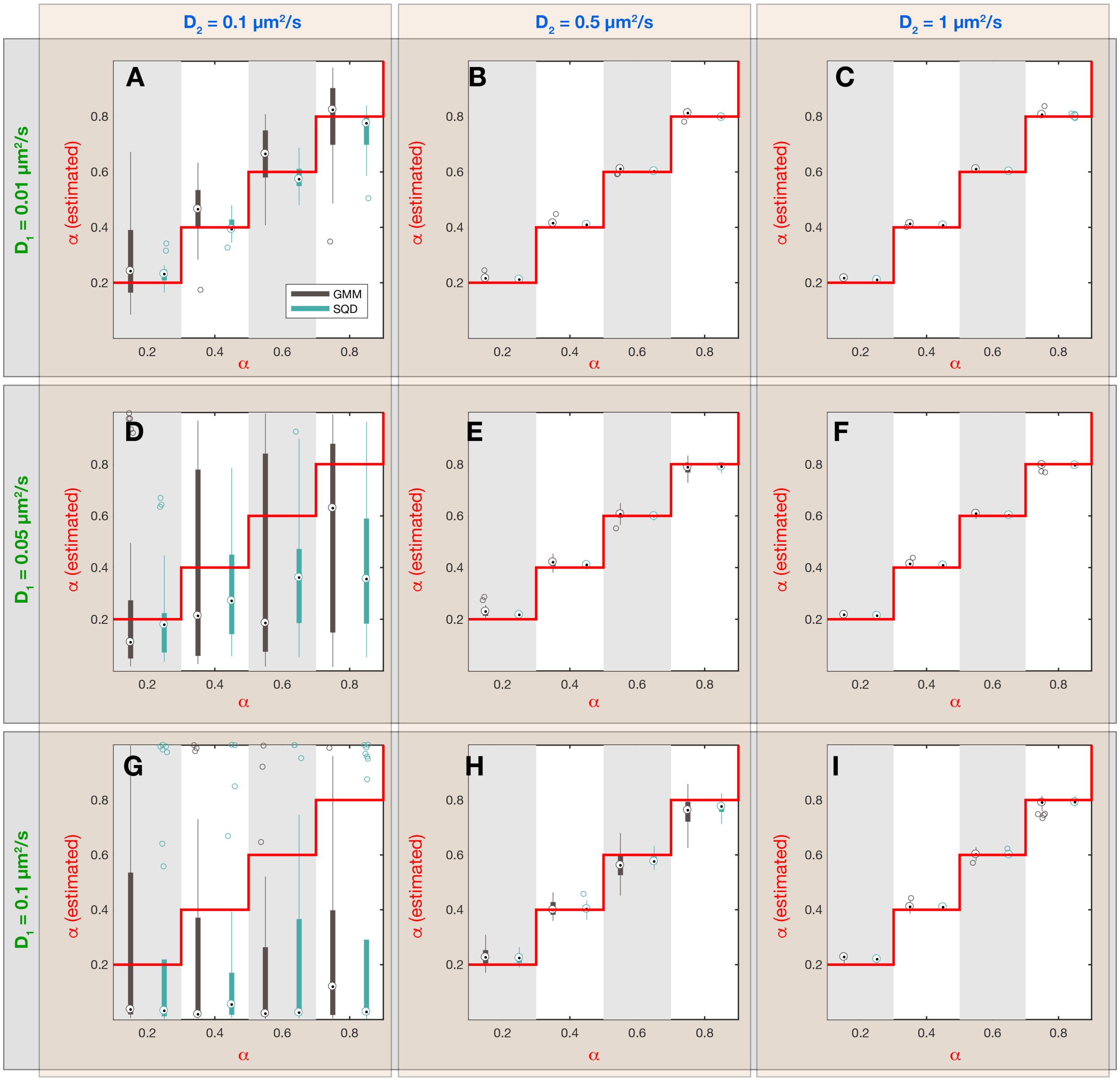

Figure 10 | Dependence of estimated fraction α of the slow subpopulation on simulation parameters and inference method. Here, the SQD method was applied for only single frame displacements (*t=1Δτ*), showing that SQD and GMM methods lead to equivalent results under these inference parameters.

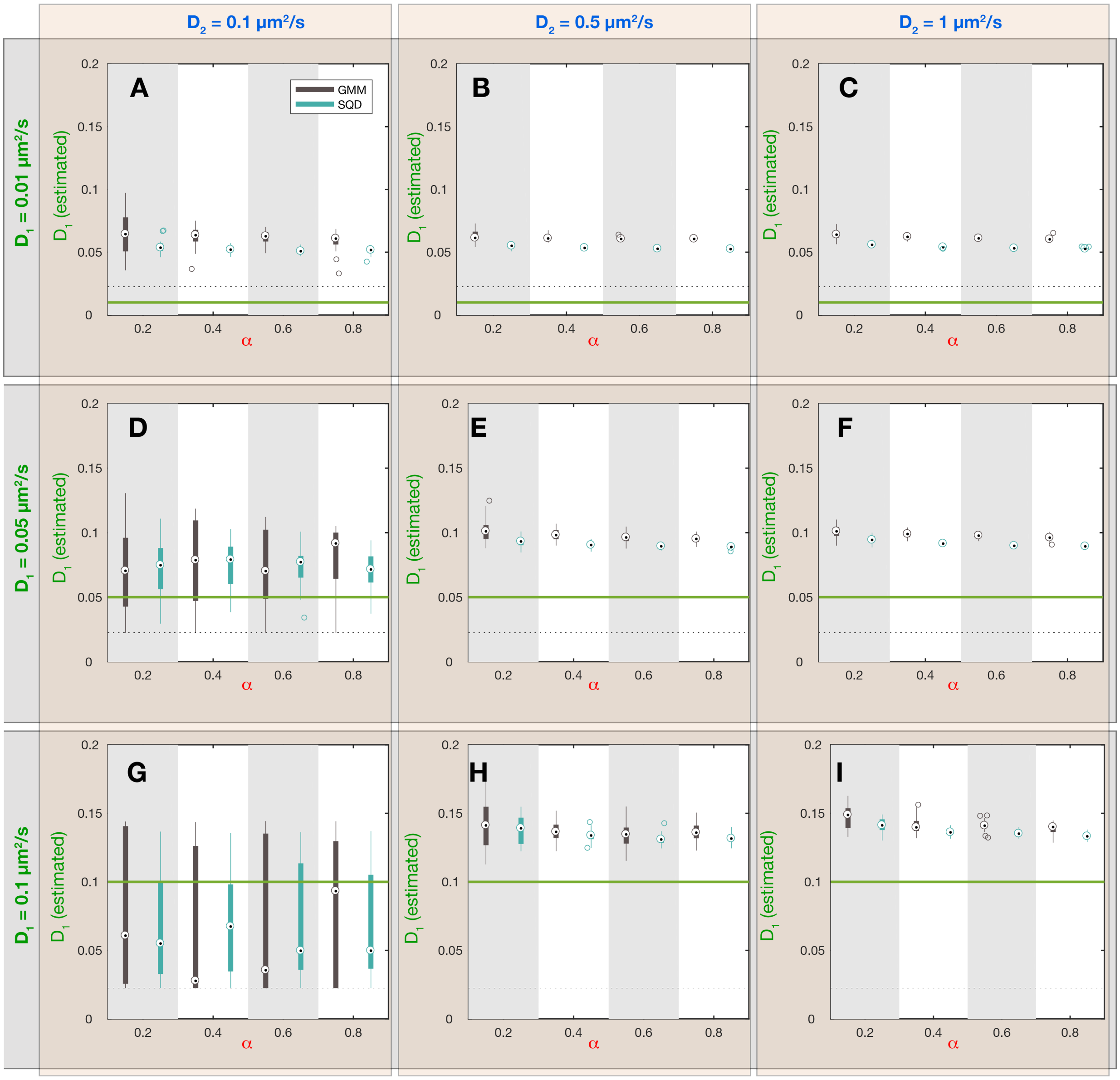

Figure 11 | Dependence of estimated diffusion constant D_1 (slow subpopulation) on simulation parameters and inference method. Here, the SQD method was applied for only single frame displacements ($t=1\Delta\tau$), showing that SQD and GMM methods lead to equivalent results under these inference parameters.

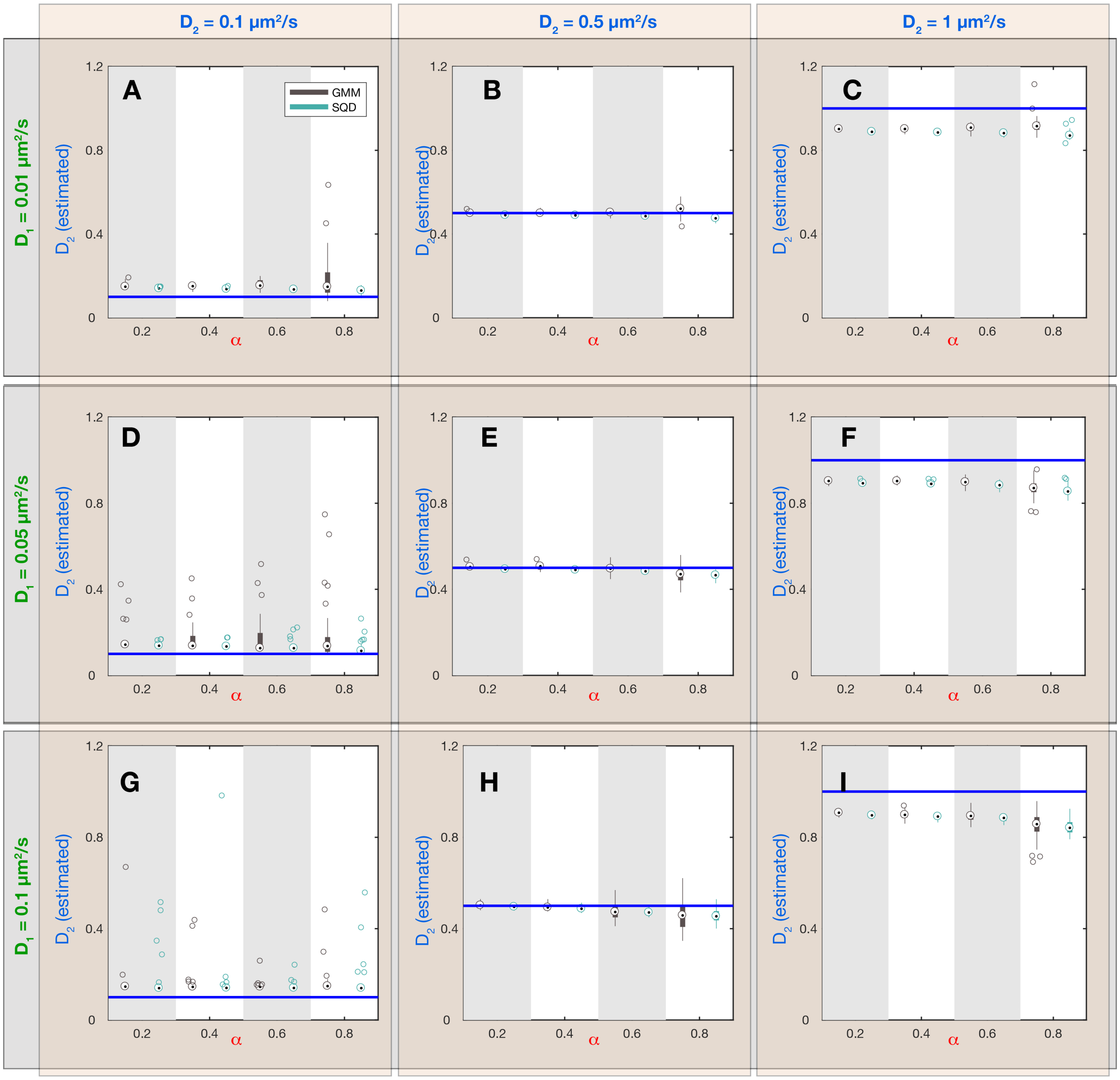

Figure 12 | Dependence of estimated diffusion constant *D_2* (fast subpopulation) on simulation parameters and inference method. Here, the SQD method was applied for only single frame displacements ($t=1\Delta\tau$), showing that SQD and GMM methods lead to equivalent results under these inference parameters.
